# The Regulatory Effects of Acetyl-CoA Distribution in the Healthy and Diseased Brain

**DOI:** 10.3389/fncel.2018.00169

**Published:** 2018-07-10

**Authors:** Anna Ronowska, Andrzej Szutowicz, Hanna Bielarczyk, Sylwia Gul-Hinc, Joanna Klimaszewska-Łata, Aleksandra Dyś, Marlena Zyśk, Agnieszka Jankowska-Kulawy

**Affiliations:** Department of Laboratory Medicine, Faculty of Medicine, Medical University of Gdańsk, Gdańsk, Poland

**Keywords:** acetyl-CoA, acetylcholine, *N*-acetyl-L-aspartate, nerve growth factor, protein acetylations, metabolic compartmentation, neuronal metabolism, neurodegeneration

## Abstract

Brain neurons, to support their neurotransmitter functions, require a several times higher supply of glucose than non-excitable cells. Pyruvate, the end product of glycolysis, through pyruvate dehydrogenase complex reaction, is a principal source of acetyl-CoA, which is a direct energy substrate in all brain cells. Several neurodegenerative conditions result in the inhibition of pyruvate dehydrogenase and decrease of acetyl-CoA synthesis in mitochondria. This attenuates metabolic flux through TCA in the mitochondria, yielding energy deficits and inhibition of diverse synthetic acetylation reactions in all neuronal sub-compartments. The acetyl-CoA concentrations in neuronal mitochondrial and cytoplasmic compartments are in the range of 10 and 7 μmol/L, respectively. They appear to be from 2 to 20 times lower than acetyl-CoA Km values for carnitine acetyltransferase, acetyl-CoA carboxylase, aspartate acetyltransferase, choline acetyltransferase, sphingosine kinase 1 acetyltransferase, acetyl-CoA hydrolase, and acetyl-CoA acetyltransferase, respectively. Therefore, alterations in acetyl-CoA levels alone may significantly change the rates of metabolic fluxes through multiple acetylation reactions in brain cells in different physiologic and pathologic conditions. Such substrate-dependent alterations in cytoplasmic, endoplasmic reticulum or nuclear acetylations may directly affect ACh synthesis, protein acetylations, and gene expression. Thereby, acetyl-CoA may regulate the functional and adaptative properties of neuronal and non-neuronal brain cells. The excitotoxicity-evoked intracellular zinc excess hits several intracellular targets, yielding the collapse of energy balance and impairment of the functional and structural integrity of postsynaptic cholinergic neurons. Acute disruption of brain energy homeostasis activates slow accumulation of amyloid-β_1-42_ (Aβ). Extra and intracellular oligomeric deposits of Aβ affect diverse transporting and signaling pathways in neuronal cells. It may combine with multiple neurotoxic signals, aggravating their detrimental effects on neuronal cells. This review presents evidences that changes of intraneuronal levels and compartmentation of acetyl-CoA may contribute significantly to neurotoxic pathomechanisms of different neurodegenerative brain disorders.

## Introduction

Cellular diversity is a principal morphologic and functional property of the brain. It includes the existence of different classes of neuronal, astroglial, microglial, and oligodendroglial cells, and also several supporting vascular and meningeal membranes cells. The neurons constitute a relatively small but highly heterogenous, and not evenly distributed, fraction of the whole brain cell population (Herculano-Houzel, 2014). Their principal function neurotransmission includes synthesis, vesicular accumulation, and quantal release of diverse neurotransmitters and regulatory compounds. The former, when released in quantal mode from depolarized axonal terminals, may exert either activatory or inhibitory effects on postsynaptic parts of recipient neurons. Continuous generation of action potentials and restoration of resting membrane potentials with a 5–50 Hz frequency, requires high rates of energy production. These two neuronal parameters may be traced *in vivo* by electroencephalography and CT-PET-MRI functional imaging (Jagust et al., 2015). Three dimensional mapping and dynamic studies of regional^18^F-deoxyglucose uptake, or changes in phosphocreatine, ATP, *N-*acetyl-L-aspartate (NAA) or lactate levels may provide a precise picture of energy metabolism in each selected anatomical structure of the brain. They also provide an accurate localization and analysis of metabolic disturbances in diverse brain pathologies (Kochunov et al., 2010; Kato et al., 2016; Zhong et al., 2014). Several reports indicate that shifts in cellular compartmentation and rates of metabolism of direct energy precursors such as pyruvate/lactate and its conversion to acetyl-CoA by PDHC may take part in the adaptative processes during brain maturation, aging, and diverse neuropathophysiological conditions (Halim et al., 2010; Jha et al., 2016). Hence, acetyl-CoA as an immediate substrate for TCA and diverse key acetylation reactions should be considered as one of the key regulatory signals in these conditions (Szutowicz et al., 2013, 2017; Pietrocola et al., 2015; Peng et al., 2016).

## Acetyl-CoA Precursors in the Brain

### Glucose and Derived Metabolites as a Principal Source of Acetyl-CoA and Energy in the Brain

Neurons contribute to 50–80% of overall energy balance of the whole brain using glucose oxidative metabolism as a principal energy source. Its adequate provision is assured by the presence of the high capacity medium affinity GLUT1 transporter (Km ∼ 8.0 mM) on the blood brain barrier and the high affinity GLUT3 transporter (Km ∼ 2.8 mM) on neuronal plasma membranes, respectively (Simpson et al., 2007). They may secure an adequate glucose supply even at serious hypoglycemic conditions of 2 mM range. In addition, the expression of GLUT1 transporter is inversely regulated by glycemia adapting the brain to chronic hyper or hypoglycemic conditions. Such homeostatic mechanisms stabilize glucose availability in brain extracellular compartments. The high energy demand in neurons results from their neurotransmitter functions linked with continuous depolarization/repolarization cycles of 5–50 Hz frequency. The maintenance of this basal neuronal function requires marked energy expenses for the restoration of membrane potential and preservation of the stable neurotransmitter pool in synaptic vesicles. Therefore, neurons are more susceptible than glial cells to diverse range pathologic inputs that limit the supply of oxygen and/or glucose and lactate as principal energy substrates (Szutowicz et al., 2013, 2017).

On the other hand, glial cells – that outnumber neurons depending on the region by 10 to 1 – produce only ca. 30–40% of the overall brain energy pool, utilizing a 50% fraction of supplied glucose (Jolivet et al., 2009; Herculano-Houzel, 2014). This divergence is caused by the fact that glial cells, mainly astrocytes, are net producers of lactate due to the relative prevalence of glycolysis over oxidative metabolism. The lactate released from the glial cells is taken up by neurons through MCT2 monocarboxylate transporters, of high affinity to lactate and pyruvate with Km values equal to 0.5 and 0.1 mM, respectively (Pérez-Escuredo et al., 2016). Reuptake of lactate by astroglia is prevented due to the presence of low affinity MCT4 transporters of Km about 25 mM. Thereby, lactate may be complementary to the glucose source of intraneuronal pyruvate, which in mitochondria is metabolized by PDHC, yielding acetyl-CoA. The latter is an energy precursor directly feeding the TCA cycle through citrate synthase step. In fact cultured neuronal cells grow and function well with pyruvate/lactate as the only energy substrates in the medium (Wohnsland et al., 2010). On the other hand, there are indications that *in vivo* both neurons and astroglia produce an excess of lactate, making the net contribution of extracellular lactate to the neuronal energy metabolism non-significant (Mangia et al., 2011). Astroglial cells also synthesize and release large amounts of L-glutamine, which is taken up by adjacent neurons. In glutamatergic and GABA-ergic neurons it is converted by phosphate activated glutaminase (EC 3.5.1.2) and glutamate decarboxylase (EC 4.1.1.15) to neurotransmitters: glutamate and γ-aminobutyrate (GABA), respectively.

In astrocytes, a fraction of glutamate, after conversion to α-ketoglutarate by glutamate dehydrogenase (EC 1.4.1.2)/aspartate aminotransferase (EC 2.6.1.1) reactions, may enter the TCA cycle at the KDHC step (Waagepetersen et al., 2007). In neurons, this pathway is much less active. Therefore, in neurons the metabolic flux of pyruvate through PDHC remains a key factor that determines the availability of acetyl-CoA for energy production in mitochondrial compartment and maintenance of their viability (Szutowicz et al., 1996, 2013). In accordance with this, the activities of PDHC in the brain homogenates and isolated mitochondria were found to be 4 – 10 times higher than in respective fractions of non-excitable tissues (Szutowicz, 1979; Szutowicz and Łysiak, 1980; Tomaszewicz et al., 2003; Bielarczyk et al., 2015). Also, rates of glucose uptake, glycolysis and pyruvate utilization in brain neurons are significantly higher than in astroglial, microglial, or oligodendroglial cells (Herculano-Houzel, 2011; Mangia et al., 2011; Klimaszewska-Łata et al., 2015). These findings are compatible with lower rates of overall oxidative metabolism in the glial than in the neuronal compartment (Thevenet et al., 2016). Therefore, physiologic and pathologic alterations of overall brain energy metabolism, observed in CT-PET-MRI as images of phosphocreatine, or NAA, reflect those taking place mainly in the neuronal compartments (Kochunov et al., 2010).

### Beta-Hydroxybutyrate/Acetoacetate and Brain Acetyl-CoA

Brain cells are also capable utilizing β-hydroxybutyrate/ acetoacetate (β-HB, AcAc) as a complementary source of acetyl-CoA both for energy production in mitochondria and for cytoplasmic synthetic pathways. Under physiologic non-fasting conditions their plasma concentrations are below 0.05 mM, which precludes their effective transport by MCT2 transporter, as its Kms for these metabolites are about 1 mM (Pérez-Escuredo et al., 2016). Therefore, at similar concentrations the uptake and rate of β-HB metabolism are 5 times slower than those of pyruvate. However, in a starvation or diabetic ketoacidosis brain, the levels of β-HB may rise up to 5 and higher millimolar concentrations. In such conditions β-HB may enter the brain cell mitochondria, being effectively metabolized to acetyl-CoA through β-hydroxybutyrate dehydrogenase (HBDH, EC 1.1.1.30), 3-oxoacid CoA-transferase (EC 2.8.3.5.), and acetoacetyl-CoA thiolase (EC 2.3.1.9) pathways (Buckley and Williamson, 1973; Chechik et al., 1987). In brain nerve terminals, β-HB in concentrations of 2.5–20.0 mM could supply up to a 30% pool of acetyl-CoA required for energy production and diverse synthetic pathways, including ACh synthesis (Szutowicz et al., 1994b, 1998b). In such conditions, β-HB slightly reduced pyruvate utilization due to competition for MCT2 transporter (Szutowicz et al., 1994b; Pérez-Escuredo et al., 2016). However, it did not affect glucose oxidation (McKenna, 2012). Therefore, under ketonemic conditions β-HB may support glucose in the maintenance of the proper level of acetyl-CoA in the brain (Szutowicz et al., 1994b, 1998b; Simpson et al., 2007).

**FIGURE 1:**
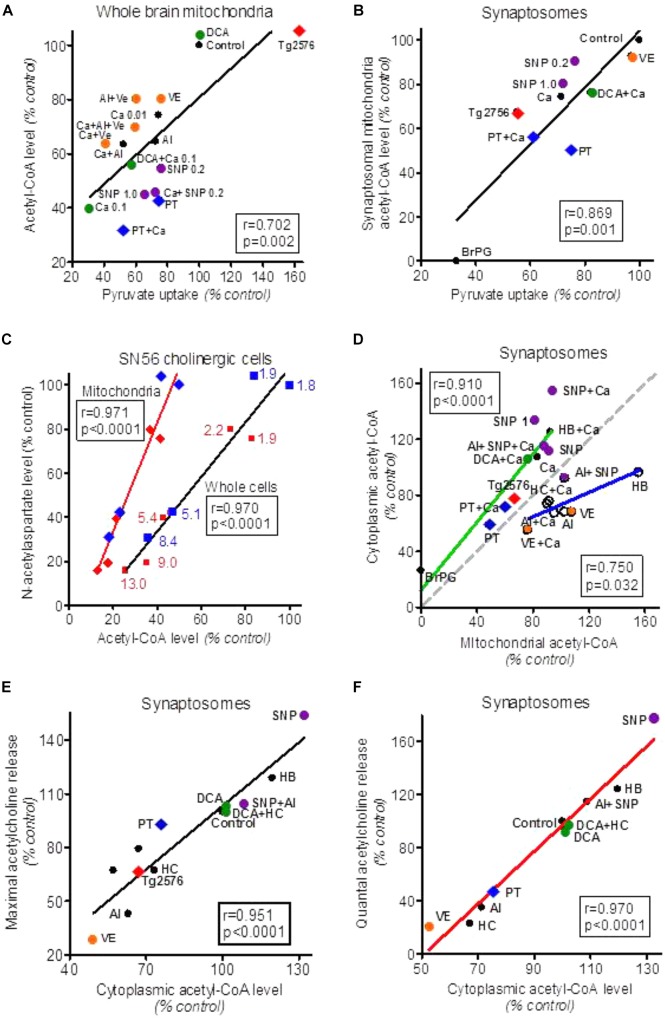
Alterations of acetyl-CoA levels in forebrain cellular compartments in different experimental models of neurotoxicity and neurodegeneration. **(A)** Effects of variations in pyruvate uptake on acetyl-CoA level in whole brain (predominately glial) mitochondria treated with different cytotoxic and cytoprotective compounds. **(B)** Effects of alterations in pyruvate uptake by isolated brain synaptosomes on acetyl-CoA level in their mitochondria. **(C)** Effects of Zn overload of SN56 non-differentiated (blue) and differentiated (red) cholinergic neuroblastoma cells (blue and red numbers indicate intracellular Zn in nmol/mg protein) on acetyl-CoA level in whole cells and cell’s mitochondria and their *N*-acetylaspartate content. **(D)** Correlations between cytoplasmic and mitochondrial acetyl-CoA levels in synaptosomes treated with cytotoxic/cytoprotective agents increasing (green plot) or decreasing/not affecting mitochondrial membrane permeability (blue plot). The gray dotted line corresponds to theoretical stoichiometric compartmentalization of acetyl-CoA. **(E)** Effect of alterations in synaptoplasmic acetyl-CoA level on maximal Ca**^++^**/K^+^ depolarization-evoked acetylcholine release/synthesis in synaptosomes treated with different cytotoxic and cytoprotective compounds. **(F)** Effect of alterations in synaptoplasmic acetyl-CoA level on Ca-dependent (quantal) acetylcholine release by synaptosomes treated with different cytotoxic and cytoprotective compounds. Al, aluminum 0.25 mmol/L; BrP, 3-bromopyruvate 0.25 mmol/L; Ca, Calcium 0.01 and 0.1 with mitochondria, Ca 1.0 mmol/L with synaptosomes; DCA, dichoroacetate 0.05 mmol/L; HB, β-hydroxybutyrate 20 mmol/L; HC, (–) hydroxycitrate 1.0 mmol/L; PT, pyrythiamin-thiamin deficient synaptosomes; SNP, sodium nitroprusside 0.2 and 1.0 mmol/L; Tg2576, transgenic AD mice; VE, verapamil 0.1 mmol/L. Data were recalculated to relative values from original data: Bielarczyk and Szutowicz (1989), Szutowicz et al. (1994a, 1998a), Tomaszewicz et al. (1997), Bielarczyk et al. (1998, 2015), Jankowska-Kulawy et al. (2010), Zyśk et al. (2017).

In fact β-HB markedly increased the level of acetyl-CoA in the mitochondrial compartment of brain nerve terminals (**Figure 1B**) (Szutowicz et al., 1998b). Synaptosomes from the brains of diabetic-ketonemic rats displayed higher levels of acetyl-CoA and rates of ACh synthesis (Szutowicz et al., 1994b, 1998b). β-HB may also prevent death of glucose deprived cultured primary cortical neurons, in energy independent mode through acetylation-induced degradation of LC3-II/p62 proteins in activated autophagosomes (Camberos-Luna et al., 2016). In high concentration β-HB also increased oxygen consumption, ATP levels, histone acetylation, and BDNF expression in cultured primary neurons from mice brain, yielding an increase of their resistance to oxidative stress (Marosi et al., 2016). Recent studies demonstrated that dietary feeding of 3xTg mice with β-HB increased the level of acetyl-CoA, accompanied by increases in NAA, citrate, and other TCA intermediates in hippocampus along with an improvement in behavioral tests (Pawlosky et al., 2017).

PET-MRI studies revealed that, in contrast to glucose, AcAc metabolism is not altered in the brains of AD patients (Cunnane et al., 2011; Castellano et al., 2015). Thus, the apparent neuroprotective effects of this ketoacid could result from the supply of acetyl-CoA by pathway bypassing glycolysis and PDHC, which are impaired in AD and other neurodegenerative diseases (Szutowicz et al., 1996, 1998b, 2013; Bubber et al., 2005; Yao et al., 2009; Jankowska-Kulawy et al., 2010; Camberos-Luna et al., 2016; Kato et al., 2016; Pawlosky et al., 2017). The ketone-evoked support of acetyl-CoA/energy metabolism may explain their protective effects against amyloid toxicity in brains of PDGFB-APPSwInd AD mice (Yin et al., 2016). These findings indicate that intermittent metabolic glucose to β-HB switching, linked with fasting-feeding cycles, may promote neuroplasticity and resistance to neurodegeneration (Mattson et al., 2018). This would be compatible with the thesis that caloric restriction – known to lengthen the lifespan of several species – may be mediated through β-HB increasing supplementation of NAPH, and expression of antioxidant enzymes (Veech et al., 2017). Clinical studies have revealed that caloric restriction, which induces ketonemia, was accompanied by slight improvements of cognitive functions in elderly people with mild cognitive impairment (Krikorian et al., 2012). Such mechanism may also contribute to the ability of β-HB to calm neuronal spiking in epileptic brains, and decrease the incidence of subclinical epileptiform activity in AD patients (Vossel et al., 2016).

In glial cells, activities of HBDH and acetoacetyl-CoA thiolase were found to be two–threefold higher than in neurons (Chechik et al., 1987). Therefore, in ketonemic conditions these ketoacids may became a significant precursor of intramitochondrial acetyl-CoA in non-neuronal cellular compartments of the brain. These data indicate that irrespective of origin, acetyl-CoA is a central point of energy metabolism in cell mitochondria and a primary precursor for multiple synthetic and signaling pathways in various extramitochondrial compartments (Szutowicz et al., 2013, 2017).

The level of acetyl-CoA in every cell type, including neurons and neuroglia, is resultant of rates of its synthesis and utilization within mitochondria and outward transport for consumption in a broad range of synthetic pathways taking place in extramitochondrial compartments (Szutowicz et al., 1996, 2013, 2017; Kouzarides, 2000; Pietrocola et al., 2015; Drazic et al., 2016).

### Acetate and Brain Acetyl-CoA

Acetate has to be reactivated by acetyl-CoA synthase (ACS, EC 6.2.1.1.). Oligodendroglia expresses acetyl-CoA synthases 1 and 2 that are located in cytoplasmic and mitochondrial compartments. They may resynthesize acetyl-CoA, supplying it directly for myelin lipids synthesis/protein acetylations, and energy production, respectively (Reijnierse et al., 1975; Szutowicz et al., 1982; Moffett et al., 2013). Low activities of ACS in nerve terminals and relatively high ones in whole brain mitochondrial fractions point to neuroglia as a principal acetate utilizing compartment of the brain (Szutowicz, 1979; Szutowicz et al., 1982; Moffett et al., 2013). However, the rate of acetate uptake was found to be independent of astroglia activity. It would suggest no direct modulating contribution of acetate to the energy metabolism of astroglial cells (Rowlands et al., 2017). On the other hand, substantial amounts of endogenous acetate may be formed by hydrolysis of acetyl-CoA, which is generated by PDHC from pyruvate derived from glucose or lactate (Łysiak et al., 1976; Rae et al., 2012). Such a thesis is also supported by the fact that over 80% of brain acetyl-CoA hydrolase (EC 3.1.2.1) activity is located in whole brain mitochondria and synaptosomal mitochondria fractions (Szutowicz et al., 1980, 1982). In fact, whole brain mitochondria utilizing pyruvate without provision of oxaloacetate, released significant amounts of acetate (Łysiak et al., 1976). On the other hand, low *V*_max_ and high acetyl-CoA Km of cytoplasmic acetyl-CoA hydrolase may suggest very slow metabolic flux through this catabolic pathway (Prass et al., 1980; Hovik et al., 1991; Suematsu and Isohashi, 2006). Nevertheless, intramitochondrially generated acetate, after being transferred to the cytoplasm, could be used by chromatin-bound ACS2 for direct acetylations of nuclear histones, yielding alterations in gene expression in brain neurons. Through such a mechanism the acetate could regulate memory consolidation in the hippocampus (Mews et al., 2017). These findings also suggest that the regulatory effects of acetate on cell metabolism occur rather *via* altering levels of acetylation of regulatory proteins than by direct influx into energy generating pathways (Rowlands et al., 2017).

### Citrate and Brain Acetyl-CoA

One should stress that in brain mitochondria the rate of acetyl-CoA utilization for citrate synthesis is about two orders of magnitude higher than the rate of its hydrolysis when comparing *V*_max_ values of citrate synthase and acetyl-CoA hydrolase, respectively (**Table 1**) (Wlassics et al., 1988; Suematsu and Isohashi, 2006). Also, isolated whole brain mitochondria or synaptosomes utilizing pyruvate with malate, accumulated several times greater amounts of citrate than those of acetate (Łysiak et al., 1976). Citrate is released from mitochondria to the cytoplasm by the citrate-malate antiporter mechanism, where it is converted back to acetyl-CoA through ATP-citrate lyase reaction (ACL, EC 2.3.3.8.) (Srere, 1965; Angielski and Szutowicz, 1967; Szutowicz et al., 1981, 1996; Gnoni et al., 2009). The (-)hydroxycitrate (HC) in 1 mmol/L concentration was found to be specific, competitive to citrate inhibitor of ACL (Szutowicz et al., 1976). At 0.5 mmol/L citrate concentration, comparable with its levels in the brain, being in range of 0.2–0.4 mmol/L, HC with Ki of 3.8 μmol/L, brought about complete inhibition of brain ACL activity, without affecting other enzymes involved in acetyl-CoA and energy metabolism (Szutowicz et al., 1976; Carlsson and Chapman, 1981; Pawlosky et al., 2017). Therefore, HC was used as a selective tool for investigating the significance of the ACL pathway in maintenance of the proper level of cytoplasmic acetyl-CoA and its contribution to different extramitochondrial synthetic pathways in the brain (Patel and Owen, 1976; Szutowicz et al., 1976, 1981, 1989, 1996, 2013; Rícný and Tucek, 1981, 1982; Constantini et al., 2007).

**Table 1:** The acetyl-CoA Km values for acetyl-CoA consuming enzymes in different cellular compartments.

Enzyme	Tissue/cells	Compartment	Km (μmol/L)	Reference
Citrate synthase	Rat brain	Mitochondria	4.8	Matsuoka and Srere, 1973
Carnitine acetyltransferase	Human liver	Mitochondria	21.3	Bloisi et al., 1990
Aspartate acetyltransferase	Rat brain	Mitochondria	58	Madhavarao et al., 2003
Acetyl-CoA hydrolase	Rat liver Rat liver Rat liver	Cytoplasm Cytoplasm Peroxisomes	150 60 400	Suematsu and Isohashi, 2006 Prass et al., 1980 Hovik et al., 1991
Choline acetyltransferase	Rat brain Human brain regions Rat brain Bovine brain	Cytoplasm Cytoplasm Highly purified Highly purified	38 32–200 46.5 16.5	Rossier et al., 1977 Koshimura et al., 1988 Ryan and McClure, 1980
Sphingosine kinase 1(COX2 acetyltransferase)	Mouse brain neurons	Cytoplasm	58.2	Lee et al., 2018
Acetyl-glutamate synthetase	Rat liver	Mitochondria	600	Coude et al., 1979
Acetyl-CoA carboxylase	Rat muscles Rat adipose tissue	Cytoplasm	31.7 ± 1.5 21.5 ± 1.0	Trumble et al., 1995
Fatty acid synthase	Rat liver (purified)	Cytoplasm	4.4	Rendina and Cheng, 2005
Acetyl-CoA acetyltransferase	Rat liver	Peroxisomes Mitochondria	<200 237	Hovik et al., 1991 Middleton, 1974
3-hydroxy-3-methyl glutaryl coenzyme A synthase	Ox liver (purified)	Cytoplasm	158	Lowe and Tubbs, 1985
ER membrane acetyl-CoA transporter (AT-1)	CHO Cell culture	Endoplasmic reticulum	14	Constantini et al., 2007
Lysine acetyltransferase BACE1	CHO Cell culture	Endoplasmic reticulum	14	Puglielli (personal report)
Lysine acetyltransferase 8 KAT8 (histone 4)	Recombinant enzyme	Nucleus	1.1-4.8	Wapenaar et al., 2015
Histone acetyltransferase Tip 60	HeLa cell nuclear extract	Nucleus	2.0	Ghizzoni et al., 2012
Histone acetyltransferase p300/CPB associated factor	Recombinant from Sf21 cells	Nucleus	0.3	Balasubramanyam et al., 2003

In depolarized nerve terminals isolated from the rat forebrain, utilizing pyruvate or glucose, 1 mmol/L HC caused a ca. 30% decrease of cytoplasmic and no alterations in intramitochondrial levels of acetyl-CoA, accompanied by respective increase of citrate accumulation and a decrease of ACh synthesis (Szutowicz et al., 1976, 1981, 1994a). A comparable, although wide range of 20–50% suppressive effects, were observed in brain slices with higher 2.5–5.0 mmol/L concentrations of HC (Sterling et al., 1981; Rícný and Tucek, 1982; Gibson and Peterson, 1983). It should be stressed that at such high concentrations, HC could exert weaker unspecific inhibitory effects on other enzymes of the citrate metabolism, as well as on PDHC and phosphofructokinase (Cheema-Dhadli et al., 1973; Szutowicz et al., 1981). In addition, they represent averaged data from multiple subcellular neuronal and glial compartments of acetyl-CoA metabolism, which might respond differentially to the same experimental conditions (Rícný and Tucek, 1982; Klimaszewska-Łata et al., 2015; Zyśk et al., 2017). These findings indicate that metabolic flux through ACL step influences both acetyl-CoA availability in cytoplasmic compartment and citrate homeostasis in neuronal cells (Szutowicz et al., 1976, 1981, 1994a; Rícný and Tucek, 1982).

#### ATP-Citrate Lyase Pathway and Cholinergic Metabolism

The rates of various extramitochondrial synthetic pathways may depend directly on the acetyl-CoA level, considering Km values of respective enzymes to this substrate as a putative regulatory factor (**Tables 1, 2**). Hence, in brain synaptosomes and slices, HC brought about inhibition of ACh synthesis/release, roughly proportional to HC-evoked decreases of acetyl-CoA levels (Rícný and Tucek, 1981, 1982; Szutowicz et al., 1981, 1994a). In brains of suckling animals, HC caused non-proportionally greater, over 60% inhibition, of non-saponifiable lipids and fatty acid synthesis (Patel and Owen, 1976). These data indicate a significant role of the ACL pathway in regulation of both ACh and lipid synthesis in brain cell cytoplasmic compartments. However, differential inhibition of both synthetic pathways by HC suggests the existence of separate cytoplasmic sub-compartments in neuronal and glial cells, being lesser and more dependent on the supply of acetyl-CoA through the ACL pathway, respectively. Also HC-evoked inhibition of ACh synthesis varied regionally, being low (17%) in the hippocampus, intermediate (30%) in the caudate nucleus, and high (55%) in the septum (Rícný and Tucek, 1982; Gibson and Peterson, 1983). These data demonstrate that the fractional contribution of the ACL pathway providing acetyl-CoA for ACh synthesis may be significantly different in various regional subpopulations of brain cholinergic neurons (Sterling et al., 1981; Rícný and Tucek, 1982; Gibson and Peterson, 1983).

**Table 2:** Estimated molar concentrations of acetyl-CoA in different compartments of the brain and clonal cells of brain origin.

Experimental model	Calculated concentration (μmol/L cell water)	Reference
Rat cerebrum	7.0 ± 0.2	Schuberth et al., 1966
Rat whole brain cortex	6.6 ± 0.3	Guynn, 1976
Rat whole brain cortex	8.1 ± 0.5	Shea and Aprison, 1977
Rat whole thalamus	11.8 ± 0.8	
Rat whole hippocampus	9.2 ± 0.5	
Rat whole striatum	9.0 ± 0.5	
Rat whole cerebellum	7.8 ± 0.5	
Nucleus caudatus (slices)	5.3 ± 0.1	Rícný and Tucek, 1981
Rat whole brain newborn	4.5 ± 0.2	Mitzen and Koeppen, 1984
60 days old	2.5 ± 0.1	
Rat whole forebrain	8.5 ± 0.4	Szutowicz and Bielarczyk, 1987
Rat whole forebrain synaptosomes	7.7 ± 0.3	Bielarczyk and Szutowicz, 1989; Szutowicz et al., 1994a,b, 1998b
Rat synaptosomal mitochondria	7.5 ± 0.3	Bielarczyk et al., 1998;
Rat synaptosomal cytoplasm	6.3 ± 0.2	Tomaszewicz et al., 2003;
Rat Whole forebrain mitochondria	17.6 ± 0.5	Jankowska-Kulawy et al., 2010
Rat whole brain	3.4 ± 1.3	Pawlosky et al., 2010
Rat whole frontal cortex		Zimatkin et al., 2011
Rat ethanol susceptible	128 ± 9	
Rat ethanol resistant	99 ± 5	
Mice forebrain synaptosomes	9.5 ± 0.7	Bielarczyk et al., 2015
Mice forebrain synaptosomal mitochondria	14.1 ± 1.6	
Mice forebrain synaptosomal cytoplasm	13.5 ± 0.5	
Mice forebrain whole forebrain mitochondria	15.7 ± 0.4	
Mice neonatal brain crude mitochondria	28.5 ± 2.7	Sun et al., 2016
Rat whole brain	4.0 ± 1.5	Shurubor et al., 2017
Clonal cell lines		
SN56 cholinergic neuroblastoma	9.6 ± 0.2	Szutowicz et al., 2004, 2005;
Mitochondria	10.9 ± 0.4	Ronowska et al., 2010;
Cytoplasm	7.2 ± 0.2	Bizon-Zygmańska et al., 2011;
SHY5Y dopaminergic neuroblastoma	11.6 ± 0.9	Klimaszewska-Łata et al., 2015;
N9 microglial cells	15.9 ± 0.8	Zyśk et al., 2017
C6 astroglial cells	4.5 ± 0.4	

*Molar concentrations of acetyl-CoA in whole brain were recalculated from original data expressed as nmol/g tissue or pmol/mg protein assuming water and protein contents in whole brain being equal to 77% and 107 mg/g of tissue, respectively (Szutowicz et al., 1982; Kozler et al., 2013). In subcellular fractions recalculations of pmol/mg protein data to molar concentrations were done assuming protein concentrations 150 and 350 mg/ml of water for cytoplasmic and mitochondrial fractions, respectively, (Nolin et al., 2016).*

This specific demand for citrate as a precursor of cytoplasmic acetyl-CoA for ACh synthesis may be met due to preferential localization of ACL in cholinergic neurons (Szutowicz, 1979; Szutowicz et al., 1980, 1983; Tomaszewicz et al., 2003). There are highly significant, positive correlations between ChAT and ACL activities in cytosolic and synaptosomal fractions isolated from brain regions of variable density of cholinergic innervation (Szutowicz et al., 1980, 1982). Activities of ACL in non-cholinergic neurons, calculated from ACL/ChAT correlation plots, were found to be equal to 2–4 nmol/min/mg protein, respectively. On the other hand, the ACL activity in cholinergic neurons, calculated from regional and cholinergic lesions studies, was in the range of 40 nmol/min/mg protein (Szutowicz et al., 1982, 1983; Tomaszewicz et al., 2003). These calculations remain in accord with immunohistochemical studies demonstrating a strong co-expression of ACL with ChAT and VAChT-expressing neurons in mice hippocampus, fascial nucleus and medulla oblongata (Beigneux et al., 2004). The tight functional links of ACL with cholinergic metabolism were demonstrated by its ability to bind ataxia-related protein forming BNIP-H-ACL-ChAT complex leading to enhanced ACh release (Sun et al., 2015). In the newborn rat brain, activity of ACL in all regions was comparably high, serving as a provider of acetyl units for structural lipid synthesis during myelinisation (Szutowicz, 1979; Szutowicz and Łysiak, 1980; Dietschy and Turley, 2004). In the course of brain development ACL activity remained high in all cortical regions, in which ChAT/ACh increased due to maturation of cholinergic neurons.

On the other hand, ACL activity in the cerebellum, devoid of cholinergic elements, decreased several fold in parallel with a maturation-dependent decrease of fatty acid synthesis due to termination of myelin and plasma membrane proteo-lipid structures formation (Szutowicz, 1979; Szutowicz and Łysiak, 1980; Szutowicz et al., 1982; Dietschy and Turley, 2004). In addition, large non-cholinergic synaptosomes isolated from the brain cortex and cerebellum contained 4 – 5 times lower ACL activities than small cortical synaptosomes containing significant fraction of cholinergic elements (Kuhar and Rommelspacher, 1974; Szutowicz et al., 1983). Cultured S20 or SN56 neuronal cells expressing mature cholinergic phenotype were found to contain higher activities of ACL than non-cholinergic neuronal and micro or astroglial cells (Szutowicz et al., 1983; Klimaszewska-Łata et al., 2015). Such cholino-tropic developmental patterns of ACL would be compatible with the demand of maturing cholinergic neurons for optimal provision of acetyl-CoA for age-dependent elevations of ACh synthesis (Szutowicz and Łysiak, 1980). Such a claim is supported by studies showing significant 30% decreases of ACL and no changes in other enzymes of acetyl-CoA metabolism activities in hippocampal synaptosomes isolated from septum electro-lesioned rat brain (Szutowicz et al., 1982). Similar alterations of ACL were observed in the IgG192 saporin (cholinergic immunotoxin) lesioned brain cortex (Tomaszewicz et al., 2003). These changes remained in accord with 70–80% decays of ChAT activities and ACh synthesis indicating loss of cholinergic neurons (Szutowicz et al., 1982; Tomaszewicz et al., 2003). Rat hippocampal synaptosomes contain about 6% subfraction of cholinergic terminals (Kuhar and Rommelspacher, 1974). Therefore, one might estimate, that ACL activities in cholinergic terminals are 10–15 times higher than in non-cholinergic ones (Szutowicz, 1979; Szutowicz et al., 1982).

Thiamine deficiency (TD) in rats caused inhibition of metabolic flux of pyruvate through PDHC, reductions of ACL-dependent fractions of cytoplasmic acetyl-CoA, and quantal ACh release from their brain nerve terminals (Jankowska-Kulawy et al., 2010). When taken together, these data indicate that ACL is preferentially expressed in cholinergic neurons, where it plays a crucial role in adequate provision of acetyl-CoA to the site of ACh synthesis (Gibson and Shimada, 1980; Szutowicz et al., 1982, 1996, 2013; Tuček, 1983; Beigneux et al., 2004).

ATP-citrate lyase may form multiple discrete subdomains executing different metabolic functions in the cells. ACL forms BNIP-ACL-ChAT complex facilitating direct provision of acetyl-CoA to the site of ACh synthesis (Sun et al., 2015). ACL also constitutes extramitochondrial sub-fractions bound with microsomes and nuclear-chromatin (Linn and Srere, 1984; Zhao et al., 2016). As such, ACL could form microdomains providing acetyl-CoA directly to acetyl-CoA transporter (AT-1) located in ER membranes (Hirabayashi et al., 2013; Pietrocola et al., 2015). In the nucleus, ACL-derived acetyl-CoA could modulate gene expression through alterations in histone acetylations (Kouzarides, 2000; Mews et al., 2017). Adipocyte studies have revealed that ACL, through cytoplasmic acetyl-CoA synthesis, may suppress expression of ACS. In *ACLYgen* knock-out cells, deficits of glucose derived acetyl-CoA could be partially alleviated by upregulation of ACS2 (Zhao et al., 2016). In such conditions acetate production by brain mitochondria exceeded that of citrate. This could increase the rate of metabolic flux through upregulated ACS2 (Łysiak et al., 1976; Zhao et al., 2016). In fact early data revealed that whole brain mitochondria incubated without oxaloacetate/malate produced several times greater amounts of acetate than those of citrate (Łysiak et al., 1976).

#### Acetyl-l-Carnitine and Direct Acetyl-CoA Transporting Pathways and Cholinergic Metabolism

Acetyl-l-carnitine is another metabolite involved in indirect transfer of acetyl-CoA from mitochondria to the cytoplasm through membrane-bound carnitine acetyl-transferases (Rícný et al., 1992; Szutowicz et al., 2005). This pathway seems to be independent of other ones described above, introducing some surplus of acetyl-CoA to the cytoplasmic compartment. Thanks to such a mode of action, acetyl-l-carnitine might exert neuroprotective effects and alleviate ACh deficits under different neurotoxic conditions, suppressing acetyl-CoA synthesis (Rícný et al., 1992; Sharman et al., 2002; Szutowicz et al., 2005).

Chronic, oral application of acetyl-l-carnitine to patients in early stages of AD improved their cognitive functions and increased brain energy phosphate levels against the placebo treated group (Pettegrew et al., 1995). In cultured cholinergic neuronal cells, acetyl-l-carnitine partially overcame the detrimental effects of neurotoxic agents through a reduction of acetyl-CoA deficits (Szutowicz et al., 2005).

In depolarized cultured cholinergic SN56 cells and brain nerve terminals, the acetyl-CoA could be also transported out of mitochondria directly through Ca-activated, verapamil-sensitive, high permeability anion channels (PTP) (Bielarczyk and Szutowicz, 1989; Szutowicz et al., 1998a, 2005). The mechanism of direct acetyl-CoA output was also found to exist in whole brain mitochondria derived mainly form glial cells (**Figure 1A**) (Rícný and Tucek, 1982; Szutowicz et al., 1998a). Analysis of our past reports revealed the existence of direct relationships between cytoplasmic and mitochondrial acetyl-CoA levels in SN56 cholinergic neuronal cells (**Figure 1D**). The use of different cytotoxic and cytoprotective agents revealed the existence of two - Ca–dependent and Ca-independent mechanisms of acetyl-CoA synthesis and distribution between mitochondrial and cytoplasmic compartments in the neurons (**Figure 1D**).

These diverse acetyl-CoA transporting pathways seem to be particularly important for depolarized cholinergic neurons. In such a functional state, cholinergic nerve terminals release quanta of ACh. This requires instant reconstitution of the ACh pool in presynaptic cholinergic terminals for continuation of neurotransmitter function. It has been shown that nerve terminals are capable of assuring an adequate provision of acetyl-CoA, and maintaining stable levels of ACh and transmission rate, even during prolonged excessive activation (Birks, 1977; Tuček, 1993; Szutowicz et al., 1996). On the other hand, decreases in cytoplasmic acetyl-CoA caused by diverse neurotoxic signals resulted in proportional suppressions of ACh content and the rate of its release from depolarized brain synaptosomes and cultured cholinergic neuronal cells (**Figure 1F**) (Szutowicz et al., 1998b, 2006, 2013).

## Acetyl-CoA Levels in the Brain

Acetyl-CoA concentrations in brain compartments are usually much lower than the Km values for several acetyl-CoA utilizing enzymes (**Tables 1, 2**). In such conditions, alterations in acetyl-CoA level may play a role of the primary factor regulating *in situ* activities of acetyl-CoA utilizing enzymes and thereby flow rates through respective metabolic pathways (Tuček, 1993; Szutowicz et al., 2013, 2017; Pietrocola et al., 2015). The analysis of acetyl-CoA determinations performed within a 50-year time span in different preparations of the brain using diverse methodologies demonstrates their strikingly good intrinsic comparability (**Table 2**). In general, acetyl-CoA concentration in whole brain tissue was reported to be below 0.01 mmol/L, with intramitochondrial levels being somewhat higher than those in the cytoplasmic compartment (**Table 2**). Some data presenting several times higher acetyl-CoA concentrations in the brain may correspond to the sum of CoA-SH + acetyl-CoA levels (**Table 2**) (Zimatkin et al., 2011). There are also indications that acetyl-CoA levels in neurons may be higher than in astroglial and lower than in microglial cells (**Table 2**) (Klimaszewska-Łata et al., 2015; Zyśk et al., 2017).

**FIGURE 2:**
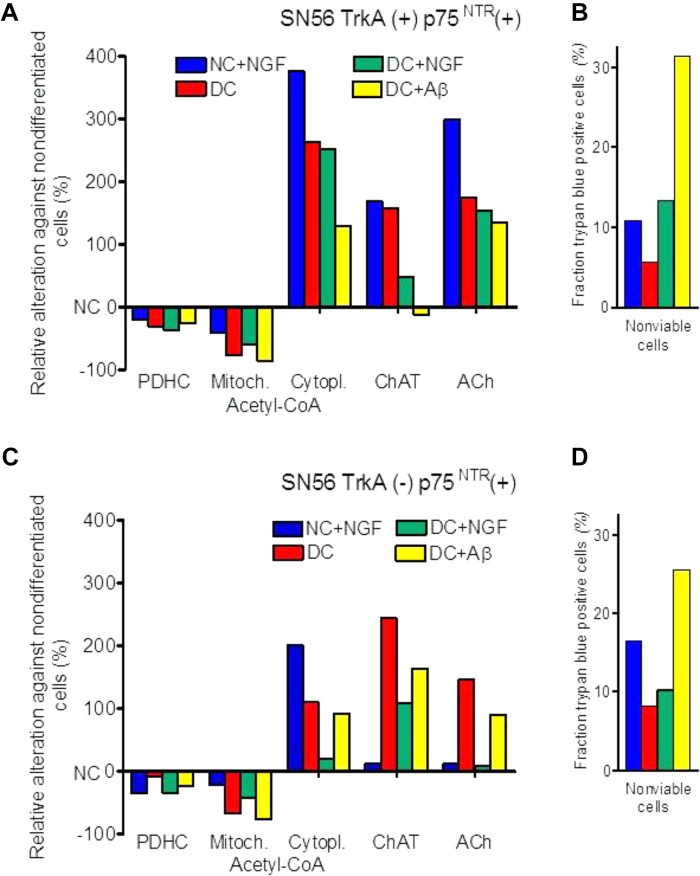
Effects of cholinergic differentiation with dibutyryl-cAMP/RA (DC) and/or with nerve growth factor (NGF) and amyloid-β (Aβ) on PDHC and ChAT activities, acetyl-CoA distribution, acetylcholine content and loss of viability of **(A,B)** cholinergic neuroblastoma SN56 with surface TrkA(+) and p75^NTR^(+) NGF receptors; **(C,D)** SN56 with TrkA(–) and p75^NTR^(+) NGF receptors. Absolute values of enzyme activities and metabolite levels corresponding to 0 ordinate are given in **Table 3**.

The heterogeneity of neuronal groups in the brain, as well as diversity of neurotransmitter systems and functions may imply differences in acetyl-CoA concentrations and pathways of its inter-compartmental redistribution (**Figure 2**) (Sterling et al., 1981; Szutowicz et al., 1981, 1996, 2017; Rícný and Tucek, 1982; Gibson and Peterson, 1983). Such a thesis is justified by studies of cholinergic neurons with high expression of the cholinergic phenotype, which displayed lower concentrations of acetyl-CoA than those with low expression of this phenotype, or non-cholinergic ones (Bielarczyk et al., 2003; Szutowicz et al., 2004, 2013; Zyśk et al., 2017). Moreover, cholinergic differentiation – despite a decrease of whole cell acetyl-CoA – was linked with its redistribution to the cytoplasmic compartment, resulting in a substrate-dependent increase in rate of ChAT reaction (**Figure 2**). It would also be compatible with the increased demand for acetyl groups for ACh synthesis in mature cholinergic neurons (Mitzen and Koeppen, 1984; Bielarczyk et al., 2003; Szutowicz et al., 2004, 2013). One may suspect that neurons of different neurotransmitter systems might contain variable profiles of intraneuronal compartmentation acetyl-CoA. Therefore, estimations of acetyl-CoA level in whole brain tissue may not adequately reflect the distribution of this metabolite between subcellular compartments in specific cell groups (**Table 2**) (Rícný and Tucek, 1982; Szutowicz et al., 1996, 2013; Pietrocola et al., 2015; Shurubor et al., 2017).

### Metabolic Regulations Through Acetyl-CoA Levels Alterations

#### Mitochondrial Acetyl-CoA, Energy Production, and Neuronal Viability

The PDHC plays a principal role in adequate provision and maintenance of the proper level of acetyl-CoA in mitochondrial compartment of neurons, platelets and, presumably, in other cell types. The enzyme is sensitive to several neurotoxic signals both under *in vivo* and *in vitro* experimental models of neurodegeneration. Inhibitors and activators of PDHC caused decreases and increases of acetyl-CoA in mitochondrial compartment of brain nerve terminals and cultured cholinergic neuroblastoma cells, respectively (**Figures 1A,B**) (Szutowicz et al., 2013, 2017). Non-toxic, low nmolar concentrations of Aβ inhibited PDHC activity *in situ* in rat hippocampal cell culture through its hyper-phosphorylation which was activated o tau protein kinase I and glycogen synthase kinase 3 (Hoshi et al., 1996, 1997). Similar conditions may occur in the brains of AD 2576Tg and 3xTg mice, in which sub micromolar Aβ accumulation was accompanied by mental impairment, suppression of pyruvate uptake, a drop of acetyl-CoA content and ACh synthesis in nerve terminals at non-altered PDHC and ChAT activities (Yao et al., 2009; Bielarczyk et al., 2015). These findings are in accord with observations of different Tg models of AD demonstrating co-existence of diverse synaptosomal mitochondrial dysfunctions as well as cholinergic and/or behavioral deficits with amyloidosis (Perez et al., 2007; Rhein et al., 2009; Webster et al., 2014; Wang et al., 2016). On the other hand, no alterations of PDHC and KDHC activities, but an increase of pyruvate uptake, was observed in whole brain mitochondria originating predominately from non-neuronal cells (Bielarczyk et al., 2015). For the above reasons, the observations made on whole brain tissue may overlook the unique alteration of pyruvate metabolism in individual cellular compartments of the brain.

There is general agreement that the increase of Zn in postsynaptic neurons of gluzinergic synapses is one of earliest events of neurodegeneration (Sensi et al., 2009; Granzotto and Sensi, 2015). This cation, in combination with Ca shifts, is considered to be a critical factor in excitotoxic cascade yielding excessive production of NOS and ROS resulting in inhibition of PDHC and diverse enzymes of energy metabolism (Ronowska et al., 2007, 2010; Granzotto and Sensi, 2015; Zyśk et al., 2017). Common cytotoxic signals such as hypoxia/ischemia and neuroinflammation may inhibit PDHC activity both in astroglial and neuronal cells through activation of PDH kinase. Aging was found to activate c-JUN-N-terminal kinase, which inhibits PDHC activity through phosphorylation of E1α subunit leading to a decrease of ATP levels in aged brains (Zhou et al., 2009). The suppression of oxidative decarboxylation of pyruvate may result in hyperlactatemia, lactate was found to act as an intercellular signaling/messenger molecule modulating neuronal plasticity, neuron glia interactions and inflammatory pain (Halim et al., 2010; Jha et al., 2015, 2016). There is also a possibility that pyruvate may itself serve not only as an energy substrate but also as a neuroprotective agent. Chronic intraperitoneal application of large doses of pyruvate to 3xTg AD mice improved memory deficits by non-energy linked reductions of oxidative stress, hyperexcitability, and maintenance of Ca/Zn homeostasis without affecting Aβ/tau pathology (Isopi et al., 2015). Activation of brain PDHC by dichloroacetate (DCA) alleviated neurological complications and improved the survival of rats after cardiac arrest (Wang et al., 2017). There is also evidence that pyruvate derived acetyl-CoA is a direct modulator of neuronal viability. The rate of neuronal SN56 cell injury by multiple neurotoxic signals displayed strong direct correlation both with rates of pyruvate utilization and acetyl-CoA levels in neuronal mitochondria (**Figures 1A,B**) (Szutowicz et al., 2013, 2017). The exogenous high energy compounds – ATP, phosphocreatine as well as acetyl-CoA – promoted physiological processing of APP and boosted survival in the cultured human SH-SY5Y neuronal cell (Sawmiller et al., 2012).

Therefore, one may assume that decreases in uptake F^18^-deoxyglucose, binding of cholinergic ligands, and a drop of NAA level in MRI/PET images of AD patient’s brains may be directly linked with acetyl-CoA deficits (**Figure 1**) (Zhong et al., 2014; Kumar et al., 2017; Schreiner et al., 2018). The regional pattern of these changes in individual AD patients matched well with specific clinical symptoms of their cognitive deficits (Mori et al., 2012; Jagust et al., 2015). These findings are compatible with postmortem studies of AD brains, revealing decreases of PDHC and TCA enzymes as well as cholinergic markers including ChAT, high affinity choline uptake (HACU), M_2_ muscarinic autoreceptors, and VAChT (Terwel et al., 1998; Pappas et al., 2000; Bubber et al., 2005; Mufson et al., 2008; Potter et al., 2011; Jagust et al., 2015). These deficits could be also caused by direct, reversible inhibition of ChAT by Aβ oligomers, yielding cholinergic dysfunction without apparent structural impairment of neuronal terminals (Hoshi et al., 1996, 1997, Nunes-Tavares et al., 2012; Bielarczyk et al., 2015). However, some patients develop clinical symptoms of AD without accumulation of Aβ (Jagust et al., 2015). It is also apparent that ca. 80% of elderly subjects display AD-type amyloid deposits, but relatively few of them suffer from AD (Ferrer, 2012; Schreiner et al., 2018). These inconsistencies may be linked with the occurrence of different ApoE phenotypes in the AD population. Thus, 99% of AD apoE4^+^ patients were also florbetapir positive, whereas in the AD apoE4- subgroup only 60% were florbetapir positive (Jagust et al., 2015). There is also a possibility that acetyl-CoA and energy deficits may appear much earlier and trigger the onset of different cholinergic encephalopathies, including AD (Szutowicz et al., 2013; Jagust et al., 2015). However, there is no data whether acetyl-CoA alterations might be involved in phenotypic diversity of AD.

The KDHC is an enzymatic complex of relatively low activity, thereby being a rate limiting step for the TCA cycle. Its inhibition seen in AD, TD, hepatic encephalopathy, and other brain pathologies has been identified as a primary factor responsible for energy deficits in neurodegenerating brains, being extensively reviewed elsewhere (Gibson et al., 2005; Butterworth, 2009).

#### Mitochondrial Acetyl-CoA and NAA Metabolism

In each type of brain cells, a major fraction of acetyl-CoA generated in mitochondria in PDHC reaction is utilized in the TCA cycle covering 98% of the energy demand. The alterations in rate of *in situ* pyruvate oxidation may bring about respective changes in levels of acetyl-CoA in the neuronal mitochondrial compartment. Inhibition of PDHC decreased availability of this substrate for the TCA cycle, yielding a decrease of ATP levels (**Figures 1A,B**) (Zyśk et al., 2017). Alterations in cytoplasmic acetyl-CoA levels correlated with its level and outward transport from the mitochondrial compartment as well as with viability of cholinergic neuronal cells (Szutowicz et al., 2013; Zyśk et al., 2017) (**Figure 1D**). In addition, 1–3% fraction of neuronal mitochondrial pool of this metabolite pool is converted intramitochondrially by aspartate *N*-acetyltransferase (AAT, EC 2.3.1.17) to NAA (Baslow, 2007; Zyśk et al., 2017). Therefore, neurons contain 98.5% of whole brain NAA. One may assume that at whole brain concentration of this amino acid, being in the range of 10 mmol/L, its intraneuronal level is likely to be several times higher (Baslow, 2007).

*N-*acetyl-l-aspartate is transferred from axons through axo-glial contact zones to oligodendrocytes and hydrolyzed to acetate by cytoplasmic *N*-acetyl aspartate aminohydrolase (EC 3.5.1.15) (Baslow, 2007; Moffett et al., 2013). This enzyme plays a significant role in myelination during brain ontogenesis, providing acetyl units for oligodendroglial energy production and fatty acid synthesis (Francis et al., 2016). Mutations in the aspartate aminohydrolase gene result in failure of myelin formation, manifested phenotypically as congenital pediatric leukodystrophy – Canavan’s disease (Moffett et al., 2013; Francis et al., 2016). Postmortem studies of multiple sclerosis brains revealed decreased levels of NAA and acetate in gray matter from parietal and motor cortex. They suggest that mitochondrial dysfunction and reduced levels of acetyl-CoA might yield deficits of NAA in neurons, which in turn would decrease its transport to oligodendrocytes. That could result in insufficient availability of acetate compromising energy production and myelin formation in these cells (Li et al., 2013; Szutowicz et al., 2017).

Aspartate *N*-acetyltransferase, the enzyme synthesizing NAA is located exclusively in neuronal mitochondria (Baslow, 2007). Its Km values for acetyl-CoA and l-aspartate are equal to 58 and 580 μmol/L, whereas concentrations of these metabolites in the brain are estimated to be 7.5–14.0 and ca. 3500 μmol/L, respectively (**Tables 1, 2**; Perry, 1982; Madhavarao et al., 2003; Papazisis et al., 2008). In such conditions, *in situ* metabolic flux through AAT reaction is estimated to be equal to ca. 16% of its maximal activity (Zyśk et al., 2017). Therefore, *in vivo*, at saturating levels of l-aspartate, the yield of AAT reaction may depend exclusively on acetyl-CoA concentration in the neuronal mitochondrial compartment. For instance, exposition of cholinergic SN56 neuronal cells to low-toxic Zn concentration, affected neither PDHC nor AAT levels/activities, but brought about similar ca. 50% decreases of pyruvate oxidation, acetyl-CoA, and NAA levels (**Figures 1B,C**). In these conditions, decreases in NAA levels strongly correlated with reductions of acetyl-CoA levels in whole cells and in mitochondrial compartment (*p* < 0.001) (**Figure 1C**) (Zyśk et al., 2017). This would be a first direct evidence for the thesis that alterations in NAA level in MRI imaging, seen in the gray matter of a neurodegenerating brain, may reflect changes in availability of acetyl-CoA in the neuronal compartment (**Figure 1C**) (Baslow, 2007; Moffett et al., 2013; Szutowicz et al., 2013, 2017; Schreiner et al., 2018).

#### Cytoplasmic Acetyl-CoA and ACh Metabolism

The functional and structural loss of cholinergic transmitter functions in the basal forebrain is a key feature of AD pathology and also includes acetyl-CoA deficits (Gelfo et al., 2013; Nell et al., 2014; Bielarczyk et al., 2015; Pepeu and Giovannini, 2017). The level of acetyl-CoA in the cytoplasmic compartment of neuronal cells is a result of the rates of its synthesis and utilization in mitochondria and the rate of its efflux to the cytoplasm through different direct (Ca-dependent) and indirect (metabolic) mechanisms (Tuček, 1993; Szutowicz et al., 1996, 2013). Compounds altering acetyl-CoA synthesis in mitochondria and increasing their membrane permeability tend to yield higher levels of cytoplasmic acetyl-CoA (**Figure 1D**, green plot) than those not changing or inhibiting membrane permeability (**Figure 1D**, blue plot). The comparisons of Km values for acetyl-CoA against different enzymes utilizing this substrate indicate that the rates of metabolic fluxes through these metabolic steps *in situ* may be several times lower than the activities of respective enzymes estimated at saturating or suboptimal substrates concentrations (**Tables 1, 2**).

The ChAT is expressed exclusively in brain cholinergic neurons, being a biomarker of their structural integrity and capacity to synthesize neurotransmitter ACh from choline and acetyl-CoA. Acetyl-CoA Km values for brain ChAT, reported in the literature, are in the range of 35–200 μmol/L (**Table 2**) and those of choline vary from 400 to 1500 μmol/L (Rossier et al., 1977; Ryan and McClure, 1980; Koshimura et al., 1988). On the other hand, averaged concentration of free choline in the brain was assessed to be ca. 60 μmol/L (Shea and Aprison, 1973; Brunello et al., 1982; Tuček, 1983, 1985; Klein et al., 1993), and that of acetyl-CoA to be close to 7 μmol/L, respectively (**Table 2**). Kinetic studies on ChAT preparations purified from rat brain revealed that in medium containing high phosphate – low chloride concentrations, characteristic for intracellular compartment, the Km’s for acetyl-CoA and choline were equal to about 40 and 1000 μmol/L, respectively (**Table 1**) (Rossier et al., 1977; Ryan and McClure, 1980). They are several times higher than intracellular levels of these substrates (**Tables 1, 2**). Considering such concentrations and kinetic constants for those metabolites, one may calculate that metabolic flux through ChAT in cholinergic neurons *in situ* is likely be equal to ca. 0.8% of maximal velocity of the enzyme, assessed at saturating concentrations of substrates (Szutowicz et al., 1982; Tuček, 1985; Koshimura et al., 1988). Such an approximation is compatible with rates of ACh synthesis in brain synaptosomes utilizing glucose or pyruvate, corresponding to 0.75–1.24% of their maximal ChAT activity (Szutowicz and Łysiak, 1980; Szutowicz et al., 1981; Bielarczyk and Szutowicz, 1989; Bielarczyk et al., 1998, 2015; Tomaszewicz et al., 2003).

The murine cholinergic SN56 neuroblastoma cells of septal origin, contain somewhat higher intracellular concentrations of acetyl-CoA and choline, of about 10 and 250 μmol/L, respectively (**Table 2**) (Lee et al., 1993). Based on the same equation, the calculated ChAT velocity in SN56 cells *in situ* should be equal to ca. 4% of its maximal rate (Jankowska et al., 2000; Szutowicz et al., 2004, 2006). Accordingly, K/Ca-induced rates of ACh synthesis in differentiated SN56 were found to vary from 1.9 to 3.5% of maximal ChAT activities, being close to its calculated *in situ* activities (Jankowska et al., 2000; Szutowicz et al., 2004, 2006; Bielarczyk et al., 2015). In such conditions the availability of acetyl-CoA in cytoplasmic compartment may be a primary factor limiting rate of ACh synthesis through concentration-dependent regulation ChAT activity (**Figures 1E,F, 2**) (Tuček, 1983, 1985; Szutowicz et al., 2013, 2017). It is also certain that pyruvate, through PDHC step, is a main source of acetyl moieties for ACh synthesis (Lefresne et al., 1973; Gibson and Shimada, 1980; Sterling et al., 1981; Bielarczyk and Szutowicz, 1989). In fact, significant alterations in intraneuronal acetyl-CoA distribution were found to take place both in physiologic and neurotoxic conditions, differentially affecting mitochondrial synthesis, transport, and its utilization in cytoplasmic compartments of cultured neuronal cells and brain synaptosomes (**Figure 1**) (Szutowicz et al., 2013, 2017). In maturing cholinergic neurons, increases of cytoplasmic level of acetyl-CoA may combine with elevation of ChAT expression, yielding a non-proportionally higher increase in the rate of ACh synthesis. For instance, cAMP/RA-induced differentiation of cholinergic SN56 cells brought a ca. 110 and 40% increases of ChAT activity and cytoplasmic acetyl-CoA level, but a 290% activation of ACh synthesis, respectively (Bielarczyk et al., 2005). This suggests the existence of synergistic, positive cooperative interactions of independent elevations of ChAT activity and acetyl-CoA levels in the stimulation of neurotransmission in maturing cholinergic neurons (Bielarczyk et al., 2005). Studies on developing rat brains revealed that adequate provision of acetyl-CoA to cytoplasmic compartment in maturing cholinergic neurons may be supported by the increase of ACL activity (Szutowicz, 1979; Szutowicz et al., 1982).

Primary and secondary thiamine deficits disturb cholinergic transmission in the brain, impairing the cognitive and motor functions in affected individuals (Gibson et al., 1982; Butterworth, 2009; Jankowska-Kulawy et al., 2010). Thiamine-deficient rats displayed inhibition of ACh synthesis and release, despite the unchanged activities of ChAT – indicating the preservation of cholinergic neurons integrity in these conditions. Thus, TD-evoked inhibition of ACh metabolism resulted exclusively from *in situ* inhibition of pyruvate oxidation by PDHC, yielding decreased availability of acetyl-CoA in the mitochondria and its secondary deficits in the ACh synthesizing compartment. (**Figures 1A,E,F**). In these conditions, the rate of ACL-dependent fraction of ACh synthesis/release fell from 0.52 to 0.26% of maximal ChAT activity (Jankowska-Kulawy et al., 2010).

In addition, in cultured SN56 cholinergic cells acute Zn overload or amprolium-thiamine depletion caused inhibition of PDHC activity and proportional suppressions of cytoplasmic acetyl-CoA level and ACh synthesis without altering ChAT activity (**Figures 1A,E,F**) (Ronowska et al., 2010; Bizon-Zygmańska et al., 2011). In the early stages of Aβ encephalopathies, inhibition of cholinergic transmission may be brought about exclusively by deficits of acetyl-CoA within structurally preserved neurons (**Figures 1E,F**) (Hoshi et al., 1996, 1997; Cuadrado-Tejedor et al., 2013; Bielarczyk et al., 2015). Such reversible alterations may precede late structural losses of cholinergic neurons (Trushina et al., 2012; Szutowicz et al., 2013, 2017). In fact, persistent exposition of differentiated SN56 cells to Zn or NO excess, simulating chronic excitotoxic conditions, brought about their morphologic deterioration and death. Neurons surviving such treatments displayed irreversible loss of both PDHC and ChAT, yielding deficits of mitochondrial and cytoplasmic acetyl-CoA and ACh metabolism (Ronowska et al., 2007; Klimaszewska-Łata et al., 2015; Zyśk et al., 2017). In latter experiments, suppressions of acetyl-CoA/ACh metabolism were caused by combination of functional and mal-adaptative structural alterations (Szutowicz et al., 2013, 2017).

One of the consequences of acetyl-CoA deficits in neuronal cytoplasm of AD brains may be decreased activity of cytoplasmic SphK1 (Ceccom et al., 2014; Bielarczyk et al., 2015). This enzyme, besides being protein kinase, also displays cyclooxygenase 2 transacetylase activity with Km for acetyl-CoA equal to 58.2 μM. This value is several times higher than neuronal levels of this metabolite (**Tables 1, 2**) (Lee et al., 2018). Acetylation of COX2 stimulates secretion of specialized proresolving mediators, which increase phagocytosis by microglia different aberrant proteins, including Aβ. There is an inverse correlation between SphK1 activity and Aβ levels in the brains of AD patients (Ceccom et al., 2014). Hence, shortages of acetyl-CoA in cytoplasmic compartment might facilitate Aβ formation, due to decreased acetylating activity of SphK1 (Lee et al., 2018). Accumulating Aβ may further aggravate acetyl-CoA deficits, directly inhibiting PDHC activity (**Figures 2A,C**) (Hoshi et al., 1996, 1997).

#### Nerve Growth Factor and Neuronal Acetyl-CoA

Nerve growth factor (NGF) is a cholinotrophic cytokine that regulates the maturation, development, and maintenance of basal cholinergic neurons through retrograde signals mediated by high affinity specific TrkA and low affinity, non-specific p75^NTR^ receptors (Szutowicz, 2001; Chao, 2003; Szutowicz et al., 2004; Boskovic et al., 2014; Isaev et al., 2017; Latina et al., 2017). Regional distribution of NGF protein and its mRNA correlate with density of cholinergic innervation (Korsching et al., 1985). Intracerebroventricular injections of NGF to neonatal rats increased in a dose-dependent manner ChAT and HACU activities in all groups of basal forebrain cholinergic neurons (Gnahn et al., 1983; Mobley et al., 1986; Auld et al., 2001). Similar cholinotrophic effects of NGF application were observed in adult animals (Williams and Rylett, 1990). Positive cholinotrophic effects of NGF were also observed *in vitro* in cultured hippocampal slices, primary cultures of septal and striatal neurons, as well as in PC12 and SN56 cholinergic cell lines. In each case NGF elevated expression of cholinergic phenotype bio-markers including; fractional content of ChAT positive cells, ChAT, HACU, VAChT levels/activities, as well as ACh contents and rates of its release (Martinez et al., 1985; Gähwiler et al., 1987; Szutowicz, 2001; Szutowicz et al., 2004; Madziar et al., 2008; Latina et al., 2017; Morelli et al., 2017). These adaptative positive cholinotrophic effects of NGF/BDNF are executed through TrkA/TrkB - CREB dependent signaling pathway yielding increased expression of the cholinergic locus and activation of Ca-accumulation/mobilization systems (Jiang et al., 1999; Finkbeiner, 2000; Chao, 2003). Similar differentiating effects are attained with diverse differentiation protocols acting through the mechanism of CREB activation (Szutowicz, 2001; Cheng et al., 2002). Impairment of TrkA/NGF signaling was involved in pathomechanism of early presynaptic dysfunction of cholinergic neurons (Latina et al., 2017). It was found to drive Aβ accumulation in cholinergic neurons (Triaca and Calissano, 2016). NGF administration prevented excitotoxic atrophy of cholinergic basal forebrain neurons (Charles et al., 1996). Treatment of neural stem cells *in vitro* with NGF stimulated their transformation toward fully functional cholinergic neuron-like cells which after transplantation into APP/PS1 transgenic mice corrected their cholinergic and behavioral deficits (Gu et al., 2015).

The p75^NTR^ receptor is a non-specific receptor shared by all four neurotrophins (Chao, 2003). Its heterodimeric complex with TrkA increased affinity to NGF and augmented Ca influx, promoting maturation of basal cholinergic neurons (**Table 3**) (Jiang et al., 1999; Mamidipudi and Wooten, 2002; Zhang et al., 2003). On the other hand, occupancy of homodimeric form of p75^NTR^ by NGF triggered ceramide-programed cell death (Carter and Lewin, 1997; Brann et al., 2002; Mamidipudi and Wooten, 2002). The excess of p75^NTR^ signaling may aggravate cognitive deficits through phenotypic suppression of cholinergic neurons (Carter and Lewin, 1997; Mamidipudi and Wooten, 2002). *In vivo* knock down of p75^NTR^ increased ChAT activity in aging rats (Barrett et al., 2016). The reduction of p75^NTR^ expression ameliorated cognitive and cholinergic deficits in Tg2576 mice (Murphy et al., 2015).

**Table 3:** Basal parameters of acetyl-CoA and ACh metabolism in TrkA(-) and TrkA(+) cholinergic SN56 neuronal cells.

Parameter	TrkA(+p75^NTR^(+)	TrkA(-) p75^NTR^(+)
PDHC activity *(nmol/min/mg protein)*	7.25 ± 0.31	7.60 ± 0.23
Mitochondrial acetyl-CoA *(pmol/mg protein)*	15.9 ± 2.1	14.2 ± 0.4
Cytoplasmic acetyl-CoA *(pmol/mg protein)*	20.1 ± 3.9	32.4 ± 3.6
Choline acetyltransferase *(nmol/min/mg protein)*	0.178 ± 0.017	0.188 ± 0.011
Acetylcholine level *(pmol/mg protein)*	149 ± 5.0	169 ± 6.0
Non-viable cell fraction *(%)*	5.5 ± 0.5	6.3 ± 0.4

*Data for recalculations were taken from: Szutowicz (2001); Madziar et al. (2003), Szutowicz et al. (2004, 2005), Bielarczyk et al. (2005), Ronowska et al. (2007).*

Differentiation of both TrkA(+)p75^NTR^(+) and TrkA(-)p75^NTR^(+) septal cholinergic SN56 neurons with cAMP/RA increased the density of p75^NTR^ receptors, apparently promoting their homo-dimerization (Szutowicz et al., 2004, 2006; Barrett et al., 2016). Such treatment also caused morphologic maturation and elevations of ChAT activity and ACh content in both TrkA (+)p75^NTR^ (+) and TrkA (-)p75^NTR^ (+) cells (**Table 3**). This was accompanied by a shift of acetyl-CoA from the mitochondria to their cytoplasm. The increased acetyl-CoA level in the cytoplasm was compatible with increased demand for acetyl units by activated ACh synthesis (**Table 3**) (Szutowicz et al., 2004, 2005). NGF application to non-differentiated cells increased ChAT/ACh only in cells with TrkA(+) but not with TrkA(-)phenotype. Moreover, NGF added either to cAMP/RA differentiated TrkA(+) and TrkA (-)cells caused parallel suppression of cytoplasmic acetyl-CoA levels and ChAT activities, as well as an increase of non-viable cell fractions (**Figures 2B,D**). It also aggravated the cytotoxic effects of Aβ or NO excess mediated by the increased density of p75^NTR^ in DC (**Figure 2**) (Szutowicz et al., 2006). These negative alterations were alleviated by the simultaneous addition of anti-p75^NTR^ antibodies (Szutowicz et al., 2004, 2005). These data indicate that both the cytoprotective/cholino-trophic and cholino-suppressive effects of NGF may be at least in part mediated through alterations of acetyl-CoA levels in mitochondrial and cytoplasmic compartments of cholinergic neurons, respectively.

#### Endoplasmic Reticulum and Nuclear Acetylations

Extramitochondrial acetyl-CoA directly affects acetylation levels of several proteins located in separate cytoplasmic, ER, and nuclear compartments. In peripheral tissues, starvation induces the depletion of cytoplasmic acetyl-CoA and protein deacetylation, being a principal signal triggering autophagy (Mariño et al., 2014). On the other hand, the replenishment of acetyl-CoA by DCA or lipoic acid inhibited autophagy through increased protein acetylations. The acetyltransferase EP300 was required for autophagy suppression by high concentrations of acetyl-CoA (Mariño et al., 2014). Also, in the brain maintenance of a proper level of protein acetylation is achieved by diverse lysine acetyltransferases, including CREB-binding protein and F1A-associated protein p300. The latter seem to be crucial for normal neurodevelopment and cognitive processes (Valor et al., 2013). Both hypoacetylation and hyperacetylation may bring about a similar spectrum of dysfunctions and/or neurodegenerative disorders (Valor et al., 2013).

A small, yet unknown fraction of cytoplasmic acetyl-Co-A has to be sub-distributed into ER/Golgi compartments to supply acetyl units for acetylation reactions of lysine residues in hundreds of structural proteins in order to regulate their turnover and activity (Kouzarides, 2000; Pehar and Puglielli, 2013; Ceccom et al., 2014). There is no acetyl-CoA synthesizing enzymes in ER lumen. However, ACS or ACL binding to ER may facilitate the provision of acetyl-CoA into their proximity (Linn and Srere, 1984; Mews et al., 2017). Such fine compartmentalization of these enzymes may assure the efficient transport of acetyl-CoA into ER by the specific membrane transporter (AT-1), a member of multiple transporters of the SLC33 family (Jonas et al., 2010; Hirabayashi et al., 2013). It would maintain acetyl-CoA on the level sufficient for activation of transient acetylations of lysine groups of different proteins in the ER lumen by specific lysine protein acetyltransferases (Constantini et al., 2007). They would include, among others, acetylations of β-amyloid precursor protein cleaving enzyme 1 (BACE 1), low density lipoproteins receptor (LDLR) or amyloid precursor protein (APP), and tubulin (Kouzarides, 2000; Jonas et al., 2010; Wong et al., 2018). Nuclear transacetylases were found to carry regulatory acetylations of histones and transcription factors modifying neuronal phenotype, plasticity, and memory/cognitive functions (Kouzarides, 2000; Mews et al., 2017).

The true level of acetyl-CoA in ER remains unknown, nevertheless it might be close or somewhat higher than that in the cytoplasmic compartment (**Table 2**). The Km values for acetyl-CoA for AT-1 transporter in ER membranes are in the range of 14 μmol/L, being higher than cytoplasmic concentrations of this metabolite (**Tables 1, 2**; Constantini et al., 2007; Jonas et al., 2010). Therefore, the rate of acetyl-CoA influx into the ER compartment may be appropriately altered both by increases and decreases of cytoplasmic levels of this metabolite taking place during neuronal maturation or excitotoxic injury, respectively (**Figure 1**) (Szutowicz, 2001; Szutowicz et al., 2004, 2013; Constantini et al., 2007; Pehar and Puglielli, 2013).

On the other hand, the nuclear membrane seems to be fully permeable for acetyl-CoA. In addition, its provision directly to acetylation sites is thought to be conducted by nuclear subfractions of ACL and ACS2 (Wellen et al., 2009; Mews et al., 2017). The presence of nuclear PDHC was also documented by Sivanand et al. (2018). Such specific compartmentation of several acetyl-CoA producing enzymes drives preferential utilization of this metabolite for acetylations of nuclear proteins by numerous HAT (Kouzarides, 2000; Drazic et al., 2016; Sivanand et al., 2018). This process would be facilitated by the fact that affinities of nuclear HATs to acetyl-CoA were found to be very high, with Km values being in range of 0.3–4.8 μmol/L (**Table 1**) (Balasubramanyam et al., 2003; Ghizzoni et al., 2012; Wapenaar et al., 2015). Therefore, neuronal-cytoplasmic acetyl-CoA concentrations of 8–13 μmol/L may assure sub-maximal rates of metabolic fluxes through nuclear acetyltransferases (**Tables 1, 2**). Consequently, the degree of nuclear histones acetylations might be regulated rather by amount/ratios of HATs and histone deacetylases than by acetyl-CoA concentration itself.

In fact, remarkable changes in proteins acetylations take place in different pathologic and physiologic conditions. Human brain autopsy revealed that increased acetylation of tau protein in AD and chronic traumatic encephalopathy brains may precede subsequent critical phosphorylation at lysine 280 (Lucke-Wold et al., 2017). On the other hand, deficient import of acetyl-CoA into ER lumen, in haploinsufficient mice carrying point mutation (S113R) in AT-1, was also associated with neurodegeneration, susceptibility to infections and increased risk of cancer (Peng et al., 2014). In another work, the same haploinsufficiency of AT-1 alleviated brain degeneration processes in (APP_695/swe_) transgenic AD mice, but not in those with Huntington’s disease (HD, R6/2) or ALS (hSOD^G93A^) (Peng et al., 2016). This discrepancy may result from the fact that inhibition of ER acetylations improved autophagy-mediated disposal toxic protein in AD. On the other hand, removal of HD and ALS aggregates took place in the cytoplasm and could not be affected by ER acetylations (Peng et al., 2016). One of the sources of such discrepancies in these pathologies could be also variable alterations in PDHC activity, which was suppressed in AD and not altered in HD (Bubber et al., 2005; Naseri et al., 2015). That might also generate differences in acetyl-CoA availability in mitochondria yielding respective down-stream changes in extramitochondrial distribution of this metabolite (Szutowicz et al., 2013, 2017; Bielarczyk et al., 2015; Peng et al., 2016). Inhibitors of histone deacetylase could exert an indirect cytoprotective effect alleviating dysfunction of PDHC, suppressing its kinases (Naia et al., 2017). Tubastatin A, the inhibitor of histone deacetylase 6 alleviated stroke-induced infarction and functional deficits, preventing a decrease in α-tubulin acetylation evoked by occlusion of middle cerebral artery (Wang et al., 2016). Inhibition of this enzyme also prevented degeneration of pluripotent stem cells from ALS patients, increasing the level of α-tubulin acetylation and the integrity of ER and axonal transport (Guo et al., 2017).

Endogenous repair of neurons in the brain is impeded by chondroitin sulfate proteoglycans or myelin associated proteins, which were found to suppress α-tubulin acetyltransferase, thereby preventing axon regeneration and growth of primary cortical neurons (Wong et al., 2018). The reconstitution level of α-tubulin acetyltransferase by lentiviral expression or increase of Rho-associated kinase reversed tubulin acetylation and neuronal growth (Wong et al., 2018). The application of icariin, a plant flavonoid, to mice with traumatic brain injury increased the acetylation of histones and prevented loss of ChAT activity and ACh content in the hippocampus (Zhang et al., 2018). Injection of another deacetylase inhibitor trichostatin A into the hippocampal CA1 area of prenatally stressed rats prevented the development of depressive behavior and reversed the suppression of AMPA glutamate receptors mRNA in the hippocampus (Lu et al., 2017). On the other hand, excessive expression of AT1/SLC33A1 in an AT-1 Tg mouse model affected dendritic branching, spine formation, and key metabolic pathways yielding cognitive deficits and autistic-like phenotype (Hullinger et al., 2016).

## Conclusion

Pyruvate-derived acetyl-CoA and oxaloacetate are principal energy-precursor substrates feeding the TCA cycle in the brain mitochondria. A relatively small fraction of mitochondrial acetyl-CoA is utilized in a large number of diverse synthetic pathways taking place in different subcellular compartments. In addition, each type of brain neuronal and glial cells apparently possesses their own individual, unique metabolic profile of acetyl-CoA distribution, fitting their specific functions. At least four subcellular structural and functional acetyl-CoA compartments have been identified, which utilize this intermediate for synthesis of a diverse range of acetylated regulatory and signaling compounds. The concentrations of acetyl-CoA in different subcellular compartments are low and may change in a fairly broad range in the course of different physiologic and pathologic conditions. On the other hand, acetyl-CoA metabolizing enzymes display relatively low affinity to this substrate. Therefore, pathophysiologic alterations in intracellular compartmentation of acetyl-CoA may be early primary signals that deeply modify cell viability and function.

In the mitochondrial compartment of neurons, the rate of NAA synthesis by AAT correlates, with acetyl-CoA level, which also directly affects neuronal viability. The output of acetyl-CoA from mitochondrial to extramitochondrial compartments depends on the rate of its synthesis by PDHC and the capacity of transport systems in the mitochondrial membranes. In cholinergic neurons, mitochondrial levels of acetyl-CoA are lower and those in the cytoplasm are higher than in respective compartments of non-cholinergic cells. The high level of acetyl-CoA in mature cholinergic neurons cytoplasm is necessary for the maintenance rate of ACh synthesis adequate to its release. In different pathophysiological conditions, the rates of ACh synthesis and release directly correlated with the levels of acetyl-CoA in the cytoplasmic compartment of the neuron. The NGF-evoked, TrkA/p75^NTR^ dependent maturation of cholinergic neurons and their susceptibility to injury may be mediated by changes in intracellular redistribution of acetyl-CoA. The provision of acetyl-CoA to ER and nuclear sub-compartments may play a key role in the development and maintenance of neuronal cells viability through alteration of the acetylation level of lysine residues of a very large range of regulatory proteins and peptides.

This review illustrates that alterations in concentration and intracellular compartmentalization of acetyl-CoA play a significant role in direct substrate-dependent regulation of multiple acetylation reactions velocities, as well as signaling molecule changing properties of several regulatory peptides and proteins. More research is required to uncover the role of acetyl-CoA in pathomechanisms and potential therapeutic approaches to neurodegenerative diseases.

## Author Contributions

AR writing most of whole chapter acetyl-CoA precursors in the brain. AS corresponding author, writing of introduction, conclusions, coordinator, main reviewer, tables and figures preparation. HB writing of subchapters introduction to acetyl-CoA levels in the brain, mitochondrial acetyl-CoA, energy production and neuronal viability. SG-H writing of endoplasmic reticulum and nuclear acetylations. JK-Ł writing of NGF and neuronal acetyl-CoA. AD writing of cytoplasmic acetyl-CoA and ACh metabolism. MZ writing of mitochondrial acetyl-CoA and NAA metabolism. AJ-K writing of subchapter acetate and brain acetyl-CoA.

## Conflict of Interest Statement

The authors declare that the research was conducted in the absence of any commercial or financial relationships that could be construed as a potential conflict of interest.

## Abbreviations

βHB: β-hydroxybutyrate
AAT: aspartate-*N*-acetyltransferase
AcAc: acetoacetate
ACh: acetylcholine
AD: Alzheimer’s disease
ALS: amyotrophic lateral sclerosis
AT-1: acetyl-CoA transporter-1(endplasmic reticulum)
ChAT: choline acetyltransferase
ER: endoplasmic reticulum
HACU: high affinity choline transporter
HAT: histone acetyltransferase
HBDH: β-hydroxybutyrate dehydrogenase
HC: (-)hydroxycitrate
KDHC: α -ketoglutarate dehydrogenase complex
NAA: *N*-acetyl-L-aspartate
NGF: nerve growth factor
NOS: nitrogen oxide reactive species
PDHC: pyruvate dehydrogenase complex
RA: retinoic acid ROS, reactive oxygen species
SphK1: sphingosine kinase 1
TCA: tricarboxylic acid cycle
VAChT: vesicular acetylcholine transporter

## References

[B1] Angielski S. Szutowicz A. (1967). Tissue content of citrate and citrate-cleavage enzyme activity during starvation and refeeding. *Nature* 213 1252–1253. 10.1038/2131252a0 3108130

[B2] Auld D. S. Mennicken F. Day J. C. Quirion R. (2001). Neurotrophins differentially enhance acetylcholine release, acetylcholine content and choline acetyltransferase activity in basal forebrain neurons. *J. Neurochem.* 77 253–262. 10.1046/j.1471-4159.2001.00234.x 11279281

[B3] Balasubramanyam K. Swaminathan V. Ranganathan A. Kundu T. K. (2003). Small molecule modulators of histone acetyltransferase p300. *J. Biol. Chem.* 278 19134–19140. 10.1074/jbc.M301580200 12624111

[B4] Barrett G. L. Naim T. Trieu J. Huang M. (2016). In vivo knockdown of basal forebrain p75 neurotrophin receptor stimulates choline acetyltransferase activity in the mature hippocampus. *J. Neurosci. Res.* 94 389–400. 10.1002/jnr.23717 26864466

[B5] Baslow M. H. (2007). “N-acetylaspartate, and N-acetylaspartylglutamate,” in *Handbook of Neurochemistry and Molecular Biology Amino acids and peptides in the nervous system*, 3rd Edn, eds Oja S. S. Schousboe A. Saransaari P. (Berlin: Springer), 305–346.

[B6] Beigneux A. P. Kosinski C. Gavino B. Horton J. D. Skarnes W. C. Young S. G. (2004). ATP-citrate lyase deficiency in the mouse. *J. Biol. Chem.* 279 9557–9564. 10.1074/jbc.M310512200 14662765 PMC2888281

[B7] Bielarczyk H. Jankowska-Kulawy A. Gul S. Pawełczyk T. Szutowicz A. (2005). Phenotype dependent differentia effects of interleukin-1beta and amyloid-beta on viability and cholinergic phenotype of T17 neuroblastoma cells. *Neurochem. Int.* 47 466–473. 10.1016/j.neuint.2005.06.010 16122837

[B8] Bielarczyk H. Jankowska-Kulawy A. Höfling C. Ronowska A. Gul-Hinc S. Roßner S. (2015). AβPP-transgenic 2576 mice mimic cell type-specific aspects of acetyl-CoA-linked metabolic deficits in Alzheimer’s disease. *J. Alzheimers Dis.* 48 1083–1094. 10.3233/JAD-150327 26402099

[B9] Bielarczyk H. Szutowicz A. (1989). Evidence for the regulatory function of synaptoplasmic acetyl-CoA in acetylcholine synthesis in nerve endings. *Biochem. J.* 262 377–380. 10.1042/bj2620377 2818575 PMC1133274

[B10] Bielarczyk H. Tomaszewicz M. Madziar B. Čwikowska J. Pawełczyk T. Szutowicz A. (2003). Relationships between cholinergic phenotype and acetyl-CoA level in hybrid murine neuroblastoma cells of septal origin. *J. Neurosci. Res.* 73 717–721. 10.1002/jnr.10711 12929139

[B11] Bielarczyk H. Tomaszewicz M. Szutowicz A. (1998). Effect of aluminum on acetyl-CoA and acetylcholine metabolism in nerve terminals. *J. Neurochem.* 70 1175–1181. 10.1046/j.1471-4159.1998.70031175.x 9489739

[B12] Birks R. I. (1977). A long-lasting potentiation of transmitter release related to an increase in transmitter stores in a sympathetic ganglion. *J. Physiol.* 271 847–862. 10.1113/jphysiol.1977.sp012028 926023 PMC1353635

[B13] Bizon-Zygmańska D. Jankowska-Kulawy A. Bielarczyk H. Pawełczyk H. Ronowska A. Marszałł M. (2011). Acetyl-CoA metabolism in amprolium-evoked thiamine pyrophosphate deficits in cholinergic SN56 neuroblastoma cells. *Neurochem. Int.* 59 208–216. 10.1016/j.neuint.2011.04.018 21672592

[B14] Bloisi W. Colombo I. Garavaglia B. Giardini R. Finocchiaro G. Didonato S. (1990). Purification and properties carnitine acetyltransferase from human liver. *Eur. J. Biochem.* 189 539–546. 10.1111/j.1432-1033.1990.tb15520.x 2351134

[B15] Boskovic Z. Alfonsi F. Rumballe B. A. Fonseka S. Windels F. Coulson E. J. (2014). The role of p75NTR in cholinergic basal forebrain structure and function. *J. Neurosci.* 34 13033–13038. 10.1523/JNEUROSCI.2364-14.2014 25253850 PMC6608337

[B16] Brann A. B. Tcherpakov M. Williams I. M. Futerman A. H. Fainzilber M. (2002). Nerve growth factor-induced p76-mediated death of cultured hippocampal neurons is age-dependent and transduced through ceramide generated by neutral sphingomyelinase. *J. Biol. Chem.* 277 9812–9818. 10.1074/jbc.M109862200 11777929

[B17] Brunello N. Cheney D. L. Costa E. (1982). Increase in exogenous choline fails to elevate the content or turnover rate of cortical, striatal or hippocampal acetylcholine. *J. Neurochem.* 38 1160–1163. 10.1111/j.1471-4159.1982.tb05364.x 7062035

[B18] Bubber P. Haroutunian V. Fisch G. Blass J. P. Gibson G. E. (2005). Mitochondrial abnormalities in Alzheimer brain: mechanistic implications. *Ann. Neurol.* 57 695–703. 10.1002/ana.20474 15852400

[B19] Buckley B. M. Williamson D. H. (1973). Acetoacetate and brain lipogenesis: developmental pattern of acetoacetyl-coenzyme A synthetase in the soluble fraction of the brain. *Biochem. J.* 132 653–656. 10.1042/bj1320653 4724596 PMC1177633

[B20] Butterworth R. F. (2009). Thiamine deficiency-related brain dysfunction in chronic liver failure. *Metab. Brain Dis.* 24 189–196. 10.1007/s11011-008-9129-y 19067139

[B21] Camberos-Luna L. Gerónimo-Olvera C. Montiel T. Rincon-Heredia R. Massieu L. (2016). The ketone body, β-hydroxybutyrate stimulates autophagic flux and prevents neuronal death induced glucose deprivation in cortical cultured neurons. *Neurochem. Res.* 41 600–609. 10.1007/s11064-015-1700-4 26303508

[B22] Carlsson C. Chapman A. G. (1981). The effect of diazepam on the cerebral metabolic state in rats and its interaction with nitrous oxide. *Anesthesiology* 54 488–495. 10.1097/00000542-198106000-00008 7235277

[B23] Carter B. D. Lewin G. R. (1997). Neutrophins live or let die: does p75NTR decide? *Neuron* 18 187–190. 10.1016/S0896-6273(00)80259-7 9052790

[B24] Castellano C. A. Nugent S. Paquet N. Tremblay S. Bocti C. Lacombe G. (2015). Lower brain 18F-fluorodeoxyglucose uptake but normal 11C-acetoacetate metabolism in mild Alzheimer’s disease dementia. *J. Alzheimers Dis.* 43 1343–1353. 10.3233/JAD-141074 25147107

[B25] Ceccom J. Loukh N. Lauwers-Cances V. Touriol C. Nicaise Y. Gentil C. (2014). Reduced sphigosine kinase-1 and enhanced sphingosine 1-phosphate lyase expression demonstrate dysregulated shingosine 1-phosphate signaling in Alzheimer’s disease. *Acta Neuropathol. Commun.* 2:12 10.1186/2951-5960-2-12 PMC3912487 24468113

[B26] Chao M. V. (2003). Neurotrophins and their receptors: a convergence point for many signaling pathways. *Nat. Rev. Neurosci.* 4 299–309. 10.1038/nrn1078 12671646

[B27] Charles V. Mufson E. J. Friden P. M. Bartus R. T. Kordower J. H. (1996). Atrophy of cholinergic basal forebrain neurons following excitotoxic cortical lesions is reversed by intravenous administration of an NGF conjugate. *Brain Res.* 728 193–203. 10.1016/0006-8993(96)00398-8 8864482

[B28] Chechik T. Roeder L. M. Tildon J. T. Poduslo S. E. (1987). Ketone body enzyme activities in purified neurons, astrocytes and oligodendrocytes. *Neurochem. Int.* 10 95–99. 10.1016/0197-0186(87)90179-3 20501089

[B29] Cheema-Dhadli S. Halperin M. L. Leeznoff C. C. (1973). Inhibition of enzymes which interact with citrate by (-)hydroxycitrate and 1.2,3,-tricarboxybenzene. *Eur. J. Biochem.* 38 98–102. 10.1111/j.1432-1033.1973.tb03038.x 4149431

[B30] Cheng H. C. Shih H. M. Chern Y. (2002). Essential role of cAMP-response element-binding protein activation by A2A adenosine receptors in rescuing the nerve growth factor-induced neurite outgrowth impaired by blockage of the MAPK cascade. *J. Biol. Chem.* 277 33930–33942. 10.1074/jbc.M201206200 12114502

[B31] Constantini C. Ko M. H. Jonas M. C. Puglielli L. (2007). A reversible form of lysine acetylation in the ER and Golgi lumen controls the molecular stabilization of BACE1. *Biochem. J.* 407 383–395. 10.1042/BJ20070040 17425515 PMC2275071

[B32] Coude F. X. Sweetman L. Nyhan W. L. (1979). Inhibition by propionyl-coenzyme A of N-acetylglutamate synthetase in rat liver mitochondria. *J. Clin. Invest.* 64 1544–1551. 10.1172/JCI109614 500823 PMC371306

[B33] Cuadrado-Tejedor M. Cabodevilla J. F. Zamarbide M. Gomez-Isla T. Franco R. Perez-Mediavilla A. (2013). Age-related mitochondrial alterations without neural loss in the hippocampus of a transgenic model of Alzheimer’s disease. *Curr. Alzheimer Res.* 10 390–405. 10.2174/1567205011310040005 23545067

[B34] Cunnane S. Nugent S. Roy M. Courchesne-Loyer A. Croteau E. Tremblay S. (2011). Brain fuel metabolism, aging and Alzheimer’s disease. *Nutrition* 27 3–20. 10.1016/j.nut.2010.07.021 21035308 PMC3478067

[B35] Dietschy J. M. Turley S. D. (2004). Thematic review series: brain lipids. Cholesterol metabolism in the central nervous system during early development and in mature animal. *J. Lipid Res.* 45 1375–1397. 10.1194/jlr.R400004-JLR200 15254070

[B36] Drazic A. Myklebust L. M. Ree R. Arnesen T. (2016). The world of protein acetylation. *Biochim. Biophys. Acta* 1864 1372–1401. 10.1016/j.bbapap.2016.06.007 27296530

[B37] Ferrer I. (2012). Defining Alzheimer as a common age-related neurodegenerative process not inevitably leading to dementia. *Progr. Neurobiol.* 97 38–51. 10.1016/j.pneurobio.2012.03.005 22459297

[B38] Finkbeiner S. (2000). CREB couples neurotrophin signals to survival messages. *Neuron* 25 11–14. 10.1016/S0896-6273(00)80866-1 10707967

[B39] Francis J. S. Wojtas I. Markov V. Gray S. J. McCown T. J. Samulski R. J. (2016). N-acetylaspartate supports the energetic demands of developmental myelination via oligodendroglial aspartoacylase. *Neurobiol. Dis.* 96 323–334. 10.1016/j.nbd.2016.10.001 27717881 PMC5102763

[B40] Gähwiler B. H. Enz A. Hefti F. (1987). Nerve growth factor promotes development of the rat septo-hippocampal cholinergic projection in vitro. *Neurosci. Lett.* 75 6–10. 10.1016/0304-3940(87)90066-8 3574769

[B41] Gelfo F. Petrosini L. Graziano A. De Bartolo P. Burello L. Vitale E. (2013). Cortical metabolic deficits in a rat model of cholinergic basal forebrain degeneration. *Neurochem. Res.* 38 2114–2123. 10.1007/s11064-013-1120-2 23925861

[B42] Ghizzoni M. Wu J. Gao T. Haisma H. J. Dekker F. J. Zheng G. Y. (2012). 6-alkylsalicylates are selective Tip60 inhibitors and target the acetyl-CoA binding. *Eur. J. Med. Chem.* 47 337–344. 10.1016/j.ejmech.2011.11.001 22100137 PMC3399519

[B43] Gibson G. E. Barclay L. Blass J. (1982). The role of the cholinergic system in thiamin deficiency. *Ann. N. Y. Acad. Sci.* 378 382–403. 10.1111/j.1749-6632.1982.tb31213.x 7044229

[B44] Gibson G. E. Blass J. P. Beal M. F. Bunik V. (2005). The α-ketoglutarate-dehydrogenase complex: a mediator between mitochondria and oxidative stress in neurodegeneration. *Mol. Neurobiol.* 31 43–63. 10.1385/MN:31:1-3:043 15953811

[B45] Gibson G. E. Peterson C. (1983). Acetylcholine and oxidative metabolism in septum and hippocampus in vitro. *J. Biol. Chem.* 258 1142–1145. 6401714

[B46] Gibson G. E. Shimada M. (1980). Studies on metabolic pathway of the acetyl group for acetylcholine synthesis. *Biochem. Pharmacol.* 29 167–174. 10.1016/0006-2952(80)90325-1 7362632

[B47] Gnahn H. Hefti F. Heumann R. Schwab M. E. Thoenen H. (1983). NGF-mediated increase of choline acetyltransferase (ChAT) in the neonatal forebrain: evidence for a physiological role of NGF in the brain? *Brain Res.* 285 45–52. 10.1016/0165-3806(83)90107-4 6136314

[B48] Gnoni G. V. Priore P. Geelen M. J. Siculella L. (2009). The mitochondrial citrate carrier: metabolic role and regulation of its activity and expression. *IUBMB Life* 61 987–994. 10.1002/iub.249 19787704

[B49] Granzotto A. Sensi S. L. (2015). Intracellular zinc is a critical intermediate in the excitotoxic cascade. *Neurobiol. Dis.* 81 25–37. 10.1016/j.nbd.2015.04.010 25940914

[B50] Gu G. Zhang W. Li M. Ni J. Wang P. (2015). Transplantation of NSC-derived cholinergic neuron-like cells improves cognitive function in APP/PS1 transgenic mice. *Neuroscience* 291 81–92. 10.1016/j.neuroscience.2015.01.073 25681520

[B51] Guo W. Naujock M. Fumagalli L. Vandoorne T. Baatsen P. Boon R. (2017). HDAC6 inhibition reverses axonal transport defects in motor neurons derived from FUS-ALS patients. *Nat. Commun.* 8:861. 10.1038/s41467-017-00911-y 29021520 PMC5636840

[B52] Guynn R. W. (1976). Effect of ethanol on brain CoA and acetyl-CoA. *J. Neurochem.* 27 303–304. 10.1111/j.1471-4159.1976.tb01583.x 182923

[B53] Halim N. D. Mcfate T. Mohyeldin A. Okagaki P. Korotchkina L. G. Patel S. M. (2010). Phosphorylation status of pyruvate dehydrogenase distinguishes metabolic phenotypes of cultured rat brain astrocytes and neurons. *Glia* 58 1168–1176. 10.1002/glia.20996 20544852 PMC2915787

[B54] Herculano-Houzel S. (2011). Scaling of brain metabolism with fixed energy budget per neuron: implications for neuronal activity, plasticity and evolution. *PLoS One* 6:e17514. 10.1371/journal.pone.0017514 21390261 PMC3046985

[B55] Herculano-Houzel S. (2014). The glia/neuron ratio: how it varies uniformly across brain structures and species and what that means for brain physiology and evolution. *Glia* 62 1377–1391. 10.1002/glia.22683 24807023

[B56] Hirabayashi Y. Nomura K. H. Nomura K. (2013). The acetyl-CoA transporter family SLC33. *Mol. Aspects Med.* 34 586–589. 10.1016/j.mam.2012.05.009 23506891

[B57] Hoshi M. Takashima A. Murayama M. Yasutake K. Yoshida N. Hoshino T. (1997). Nontoxic amyloid beta peptide 1-42 suppresses acetylcholine synthesis. Possible role in cholinergic dysfunction in Alzheimer’s disease. *J. Biol. Chem.* 272 2038–2041. 10.1074/jbc.272.4.2038 8999897

[B58] Hoshi M. Takashima A. Noguchi K. Murayama M. Sato M. Kondo S. (1996). Regulation of mitochondrial pyruvate dehydrogenase activity by tau protein kinase I/glycogen synthase kinase 3beta in brain. *Proc. Natl. Acad. Sci. U.S.A.* 93 2719–2723. 10.1073/pnas.93.7.2719 8610107 PMC39697

[B59] Hovik R. Brodal B. Bartlett K. Osmundsen H. (1991). Metabolism of acetyl-CoA by isolated peroxisomal fractions: formation of acetate and acetoacetyl-CoA. *J. Lipid Res.* 32 993–999. 1682408

[B60] Hullinger R. Li M. Wang J. Peng Y. Dowell J. A. Bomba-Warczak E. (2016). Increased expression of AT-1/SLC33A1 causes an autistic-like phenotype in mice by affecting dendritic branching and spine formation. *J. Exp. Med.* 213 1267–1284. 10.1084/jem.20151776 27242167 PMC4925020

[B61] Isaev N. K. Stelmashook E. V. Genrikhs E. E. (2017). Role of nerve growth factor in plasticity of forebrain cholinergic neurons. *Biochemistry* 82 291–300. 10.1134/S0006297917030075 28320270

[B62] Isopi E. Granzotto A. Corona C. Bomba M. Ciavardelli D. Curcio M. (2015). Pyruvate prevents the development of age-dependent cognitive deficits in a mouse model of Alzheimer’s disease without reducing amyloid and tau pathology. *Neurobiol. Dis.* 81 214–224. 10.1016/j.nbd.2014.11.013 25434488

[B63] Jagust W. J. Landau S. M. Koeppe R. A. Reiman E. M. Chen K. Mathis C. A. (2015). The Alzheimer’s disease neuroimaging initiative 2PET Core: 2015. *Alzheimer’s Dement.* 11 757–771. 10.1016/j.jalz.2015.05.001 26194311 PMC4510459

[B64] Jankowska A. Madziar B. Tomaszewicz M. Szutowicz A. (2000). Acute and chronic effects of aluminum on acetyl-CoA and acetylcholine metabolism in differentiated and nondifferentiated SN56 cholinergic cells. *J. Neurosci. Res.* 62 615–622. 10.1002/1097-4547(20001115)62:4<615::AID-JNR17>3.0.CO;2-1 11070506

[B65] Jankowska-Kulawy A. Bielarczyk H. Pawełczyk T. Wróblewska M. Szutowicz A. (2010). Acetyl-CoA and acetylcholine metabolism in nerve terminal compartment of thiamine deficient rat brain. *J. Neurochem.* 115 333–342. 10.1111/j.1471-4159.2010.06919.x 20649840

[B66] Jha M. K. Lee I. K. Suk K. (2016). Metabolic reprogramming by the pyruvate dehydrogenase kinase-lactic acid axis: linking metabolism and diverse neuropathophysiologies. *Neurosci. Biobehav. Rev.* 68 1–19. 10.1016/j.neubiorev.2016.05.006 27179453

[B67] Jha M. K. Song G. J. Lee M. G. Jeoung N. H. Go Y. Harris R. A. (2015). Metabolic connection of inflammatory pain: pivotal role of a pyruvate dehydrogenase kinase-pyruvate dehydrogenase-lactic acid axis. *J. Neurosci.* 35 14353–14369. 10.1523/JNEUROSCI.1910-15.2015 26490872 PMC6605420

[B68] Jiang H. Takeda K. Lazarovici P. Katagiri Y. Yu Z.-X. Dickens G. (1999). Nerve growth factor (NGF)-induced calcium influx and intracellular calcium mobilization in 3T3 cells expressing NGF receptor. *J. Biol. Chem.* 274 26209–26216. 10.1074/jbc.274.37.26209 10473574

[B69] Jolivet R. Magistretti P. J. Weber B. (2009). Deciphering neuron-glia compartmentalization in cortical energy metabolism. *Front. Neuroenergetics* 1:4. 10.3389/neuro.14.004.2009 19636395 PMC2715922

[B70] Jonas M. C. Pehar M. Puglielli L. (2010). AT-1 is the ER membrane acetyl-CoA transporter and is essential for cell viability. *J. Cell Sci.* 123 3378–3388. 10.1242/jcs.068841 20826464 PMC2939804

[B71] Kato T. Inui Y. Nakamura A. Ito K. (2016). Brain fluorodeoxyglucose (FDG) PET in dementia. *Ageing Res. Rev.* 30 73–84. 10.1016/j.arr.2016.02.003 26876244

[B72] Klein J. Gonzalez R. Köppen A. Löffelholz K. (1993). Free choline and choline metabolites in rat brain and body fluids: sensitive determination and implications for choline supply to the brain. *Neurochem. Int.* 22 293–300. 10.1016/0197-0186(93)90058-D 8443570

[B73] Klimaszewska-Łata J. Gul-Hinc S. Bielarczyk H. Ronowska A. Zyśk M. Grużewska K. (2015). Differential effects of lipopolysaccharide on energy metabolism in murine microglial N9 and cholinergic SN56 neuronal cells. *J. Neurochem.* 133 284–297. 10.1111/jnc.12979 25345568

[B74] Kochunov P. Coyle T. Lancaster J. Robin D. A. Hardies J. Kochunov V. (2010). Processing speed is correlated with cerebral health markers in the frontal lobes as quantified by neuroimaging. *Neuroimage* 49 1190–1199. 10.1016/j.neuroimage.2009.09.052 19796691 PMC2789896

[B75] Korsching S. Auburger G. Heumann R. Scott J. Thoenen H. (1985). Levels of nerve growth factor and its mRNA in the central nervous system of the rat correlate with cholinergic innervation. *EMBO J.* 4 1389–1393. 2411537 10.1002/j.1460-2075.1985.tb03791.x PMC554356

[B76] Koshimura K. Nakamura S. Miwa S. Fujiwara M. Kameyama M. (1988). Regional difference in the kinetics of choline acetyltransferase in brains of neurologically normal elderly people and those with Alzheimer-type dementia. *J. Neurol. Sci.* 84 141–146. 10.1016/0022-510X(88)90119-0 3379442

[B77] Kouzarides T. (2000). Acetylation: a regulatory modification to rival phosphorylation? *EMBO J.* 19 1176–1179. 10.1093/emboj/19.6.1176 10716917 PMC305658

[B78] Kozler P. Riljak V. Pokorny J. (2013). Both water intoxication and osmotic BBB disruption increase brain water content in rats. *Physiol. Res.* 62(Suppl. 1), S75–S80. 24329706 10.33549/physiolres.932566

[B79] Krikorian R. Shidler M. D. Dangelo K. Couch S. C. Benoit S. C. Clegg D. J. (2012). Dietary ketosis enhances memory in mild cognitive impairment. *Neurobiol. Aging* 33 425.e19–425.e27. 10.1016/j.neurobiolaging.2010.10.006 21130529 PMC3116949

[B80] Kuhar M. J. Rommelspacher H. (1974). Acetylcholinesterase-staining synaptosomes from rat hippocampus: relative frequency and tentative estimation of internal concentration of free or ‘labile bound’ acetylcholine. *Brain Res.* 77 85–96. 10.1016/0006-8993(74)90806-3 4850389

[B81] Kumar R. Kumar A. Långströ M. B. Darreh-Shori T. (2017). Discovery of novel choline acetyltransferase inhibitors using structure-based virtual screening. *Sci. Rep.* 7:16287. 10.1038/s41598-017-16033-w 29176551 PMC5701137

[B82] Latina V. Caioli S. Zona C. Ciotti M. T. Amadoro G. Callisano P. (2017). Impaired NGF/TrkA signaling causes early AD-linked presynaptic dysfunction in cholinergic primary neurons. *Front. Cell. Neurosci.* 11:68. 10.3389/fncel.2017.00068 28360840 PMC5350152

[B83] Lee H. C. Fellenz-Maloney M. P. Liscovitch M. Blusztajn J. K. (1993). Phospholipase D-catalyzed hydrolysis of phosphatidylcholine provides the choline precursor for acetylcholine synthesis in human neuronal cell line. *Proc. Natl. Acad. Sci. U.S.A.* 90 10086–10090. 10.1073/pnas.90.21.10086 8234260 PMC47718

[B84] Lee J. Y. Han S. H. Park M. H. Baek B. Song I. S. Choi M. K. (2018). Neuronal SphK1 acetylates COX2 and contributes to pathogenesis in a model of Alzheimer’s disease. *Nat. Commun.* 9:1479. 10.1038/s41467-018-03674-2 29662056 PMC5902554

[B85] Lefresne P. Guyenet P. Glowinski J. (1973). Acetylcholine synthesis from (2-14C) pyruvate in rat striatal slices. *J. Neurochem.* 20 1083–1097. 10.1111/j.1471-4159.1973.tb00079.x 4697871

[B86] Li S. Clements R. Sulak M. Gregory R. Freeman E. McDonough J. (2013). Decreased NAA in gray matter is correlated with decreased availability of acetate in white matter in postmortem multiple sclerosis cortex. *Neurochem. Res.* 38 2385–2396. 10.1007/s11064-013-1151-8 24078261 PMC3880684

[B87] Linn T. C. Srere P. A. (1984). Binding of ATP citrate lyase to the microsomal fraction of rat liver. *J. Biol. Chem.* 259 13379–13384. 6490657

[B88] Lowe D. M. Tubbs P. K. (1985). 3-hydroxy-3-methylglutaryl-coenzyme A synthase from ox liver. Purification, molecular and catalytic properties. *Biochem. J.* 227 591–599. 10.1042/bj2270591 2860895 PMC1144879

[B89] Lu Y. Zhang J. Zhang L. Dang S. Su Q. Zhang H. (2017). Hippocampal acetylation may improve prenatal-stress-induced depression-like behavior of male offspring rats through regulating AMPARs expression. *Neurochem. Res.* 42 3456–3464. 10.1007/s11064-017-2393-7 29019029

[B90] Lucke-Wold B. Seidel K. Udo R. Omalu B. Ornstein M. Nolan R. (2017). Role of tau acetylation in Alzheimer’s disease and chronic traumatic encephalopathy: the way forward for successful treatment. *J. Neurol. Neurosurg.* 4:140. PMC5738035 29276758

[B91] Łysiak W. Szutowicz A. Angielski S. (1976). Pyruvate metabolism in rat brain mitochondria. *Acta Biochim. Polon.* 23 325–333. 13594

[B92] Madhavarao C. N. Chinopoulos C. Chandrasekaran K. Namboodiri M. A. (2003). Characterization of the N-acetylaspartate biosynthetic enzyme from rat brain. *J. Neurochem.* 86 824–835. 10.1046/j.1471-4159.2003.01905.x 12887681

[B93] Madziar B. Shah S. Brock M. Burke R. Lopez-Coviela I. Nickel A. C. (2008). Nerve growth factor regulates the expression of the cholinergic locus and the high-affinity choline transporter via the Akt/PKB signaling pathway. *J. Neurochem.* 107 1284–1293. 10.1111/j.1471-4159.2008.05681.x 18793330 PMC5912896

[B94] Madziar B. Tomaszewicz M. Matecki A. Bielarczyk H. Szutowicz A. (2003). Interactions between p75 and TrkA receptors in differentiation and vulnerability of SN56 cholinergic cells to beta-amyloid. *Neurochem. Res.* 28 461–465. 10.1023/A:1022800802179 12675131

[B95] Mamidipudi V. Wooten M. W. (2002). Dual role for p75(NTR) signaling in survival and cell death: can intracellular mediators provide an explanation? *J. Neurosci. Res.* 68 373–384. 10.1002/jnr.10244 11992464

[B96] Mangia S. DiNuzzo M. Giove F. Carruthers A. Simpson I. A. Vannucci S. J. (2011). Response to comment on recent modeling studies of astrocyte-neuron metabolic interactions’: much ado about nothing. *J. Cereb. Blood Flow Metab.* 31 1346–1353. 10.1038/jcbfm.2011.29 21427731 PMC3130323

[B97] Mariño G. Pietrocola F. Eisenberg T. Kong Y. Malik S. A. Andryushkova A. (2014). Regulation of autophagy by cytosolic acetyl-coenzyme A. *Mol. Cell.* 53 710–725. 10.1016/j.molcel.2014.01.016 24560926

[B98] Marosi K. Kim S. W. Moehl K. Scheibye-Knudsen M. Cheng A. Cutler R. (2016). 3-Hydroxybutyrate regulates energy metabolism and induces BDNF expression in cerebral cortical neurons. *J. Neurochem.* 139 769–781. 10.1111/jnc.13868 27739595 PMC5123937

[B99] Martinez H. J. Dreyfus C. F. Jonakait G. M. Black I. B. (1985). Nerve growth factor promotes cholinergic development in brain striatal cultures. *Proc. Natl. Acad. Sci. U.S.A.* 82 7777–7781. 10.1073/pnas.82.22.7777 3865196 PMC391417

[B100] Matsuoka Y. Srere P. A. (1973). Kinetic studies of citrate synthase from rat kidney and rat brain. *J. Biol. Chem.* 248 8022–8030. 4201777

[B101] Mattson M. P. Moehl K. Ghena N. Schmaedick M. Cheng A. (2018). Intermittent metabolic switching, neuroplasticity and brain health. *Nat. Rev. Neurosci.* 19 63–80. 10.1038/nrn.2017.156 29321682 PMC5913738

[B102] McKenna M. C. (2012). Substrate competition studies demonstrate oxidative metabolism of glucose, glutamate, glutamine, lactate and 3-hydroxybutyrate in cortical astrocytes from rat brain. *Neurochem. Res.* 37 2613–2626. 10.1007/s11064-012-0901-3 23079895 PMC3547390

[B103] Mews P. Donahue G. Drake A. M. Luczak V. Abel T. Berger S. L. (2017). Acetyl-CoA synthetase regulates histone acetylation and hippocampal memory. *Nature* 546 381–386. 10.1038/nature22405 28562591 PMC5505514

[B104] Middleton B. (1974). The kinetic mechanism and properties of the cytoplasmic acetoacetyl-coenzyme A thiolase from rat liver. *Biochem. J.* 139 109–121. 10.1042/bj1390109 4156910 PMC1166257

[B105] Mitzen E. J. Koeppen A. H. (1984). Malonate, malonyl-CoA, and acetyl-coenzyme A in developing rat brain. *J. Neurochem.* 43 499–506. 10.1111/j.1471-4159.1984.tb00927.x 6429279

[B106] Mobley W. C. Rutkowski J. L. Tennekoon G. I. Gemski J. Buchanan K. Johnston M. V. (1986). Nerve growth factor increases choline acetyltransferase activity in developing basal forebrain neurons. *Brain Res.* 387 53–62. 10.1016/0169-328X(86)90020-3 3742234

[B107] Moffett J. R. Arun P. Ariyannyr P. S. Namboodiri A. M. (2013). N-acetylaspartate reductions in brain injury: impact on post-injury neuroenergetics, lipid synthesis, and protein acetylation. *Front. Neuroenergetics* 5:11. 10.3389/fnene.2013.00011 24421768 PMC3872778

[B108] Morelli A. Sarchielli E. Guarnieri G. Coppi E. Pantano D. Comeglio P. (2017). Young human cholinergic neurons respond to physiological regulators and improve cognitive symptoms in an animal model of Alzheimer’s disease. *Front. Cell. Neurosci.* 11:339 10.3389/fncel.2017.00339 PMC5666298 29163051

[B109] Mori T. Maeda J. Shimada H. Higuchi M. Shinotoh H. Ueno S. (2012). Molecular imaging of dementia. *Psychogeriatrics* 12 106–114. 10.1111/j.1479-8301.2012.00409.x 22712644

[B110] Mufson E. J. Counts S. E. Perez S. E. Ginsberg S. D. (2008). Cholinergic system during the progression of Alzheimer’s disease: therapeutic implications. *Expert Rev. Neurother.* 8 1705–1718. 10.1586/14737175.8.11.1703 18986241 PMC2631573

[B111] Murphy M. Wilson Y. M. Vargas E. Munro K. M. Smith B. Huang A. (2015). Reduction of p75 neurotrophin receptor ameliorates the cognitive deficits in a model of Alzheimer’s disease. *Neurobiol. Aging* 36 740–752. 10.1016/j.neurobiolaging.2014.09.014 25443284

[B112] Naia L. Cunha-Oliveira T. Rodrigues J. Rosenstock T. R. Oliveira A. Ribeiro M. (2017). Histone deacetylase inhibitors protect against pyruvate dehydrogenase dysfunction in Huntington’s disease. *J. Neurosci.* 37 2776–2794. 10.1523/JNEUROSCI.2006-14.2016 28123081 PMC6596633

[B113] Naseri N. N. Xu H. Bonica J. Vonsattel J. P. Corte E. T. Park L. C. (2015). Abnormalities in the tricarboxylic acid cycle in Huntington disease and in a Huntington disease mouse model. *J. Neuropathol. Exp. Neurol.* 74 527–537. 10.1097/NEN.0000000000000197 25978848 PMC4435838

[B114] Nell H. J. Whitehead S. N. Cechetto D. F. (2014). Age-dependent effect of β-amyloid toxicity on basal forebrain cholinergic neurons and inflammation in rat brain. *Brain Pathol.* 25 531–542. 10.1111/bpa.12199 25187042 PMC8029185

[B115] Nolin F. Michel J. Wortham L. Tchelidze P. Banchet V. (2016). Stage-specific changes in water, Na +, Cl-, and K + contents of organelles during apoptosis, demonstrated by a targeted cryo correlative analytical approach. *PLoS One* 11:e0148727. 10.1371/journal.pone.0148727 26866363 PMC4807926

[B116] Nunes-Tavares N. Santos L. E. Stutz B. Brito-Moreira J. Klein W. L. Ferreira S. T. (2012). Inhibition of choline acetyltransferase as a mechanism for cholinergic dysfunction induced by amyloid-β peptide oligomers. *J. Biol. Chem.* 287 19377–19385. 10.1074/jbc.M111.321448 22505713 PMC3365976

[B117] Papazisis G. Pourzitaki C. Sardeli C. Lallas A. Amaniti E. Kouvelas D. (2008). Deferoxamine decreases the excitatory amino acid levels and improves the histological outcome in the hippocampus of neonatal rats after hypoxia-ischemia. *Pharmacol. Res.* 57 73–78. 10.1016/j.phrs.2007.12.003 18243015

[B118] Pappas B. A. Bayley P. J. Bui B. K. Hansen L. A. Thal L. J. (2000). Choline acetyltransferase activity and cognitive domain scores of Alzheimer’s patients. *Neurobiol. Aging* 21 11–17. 10.1016/S0197-4580(00)00090-7 10794843

[B119] Patel M. S. Owen O. E. (1976). Lipogenesis from ketone bodies in rat brain. Evidence for conversion of acetoacetate into acetyl-coenzyme A in the cytosol. *Biochem. J.* 156 603–607. 10.1042/bj1560603 949342 PMC1163794

[B120] Pawlosky R. J. Kashiwaya Y. Srivastava S. King M. T. Cruthfield C. Volkov N. (2010). Alterations in brain glucose utilization accompanying elevations in blood ethanol and acetate concentrations in the rat. *Alcoholism Clin. Exp. Res.* 34 375–381. 10.1111/j.1530-0277.2009.01099.x 19951290 PMC2958045

[B121] Pawlosky R. J. Kemper M. F. Kashiwaya Y. King M. T. Mattson M. P. Veech R. L. (2017). Effects of dietary ketone esters on hippocampal glycolytic and tricarboxylic acid cycle intermediates and amino acids in 3xTgAD mouse model of Alzheimer’s disease. *J. Neurochem.* 141 195–207. 10.1111/jnc.13958 28099989 PMC5383517

[B122] Pehar M. Puglielli L. (2013). Lysine acetylation in the lumen of ER: a novel and essential function under the control of the UPR. *Biochim. Biophys. Acta* 1833 686–697. 10.1016/j.bbamcr.2012.12.004 23247107 PMC3556210

[B123] Peng Y. Kim M. J. Hullinger R. O’Riordan K. J. Burger C. Pehar M. (2016). Improved proteostasis in the secretory pathway rescues Alzheimer’s disease in the mouse. *Brain* 139 937–952. 10.1093/brain/awv385 26787453 PMC4805081

[B124] Peng Y. Li M. Clarkson B. D. Pehar M. Lao P. J. Hillmer A. T. (2014). Deficient import of acetyl-CoA into the ER lumen causes neurodegeneration an propensity to infections, inflammation, and cancer. *J. Neurosci.* 34 6772–6789. 10.1523/JNEUROSCI.0077-14.2014 24828632 PMC4019794

[B125] Pepeu G. Giovannini G. M. (2017). The fate of the brain cholinergic neurons in neurodegenerative diseases. *Brain Res.* 1670 173–184. 10.1016/j.brainres.2017.06.023 28652219

[B126] Perez S. E. Dar S. Ikonomovic M. D. DeKosky S. T. Mufson E. J. (2007). Cholinergic forebrain degeneration in the APPswe/PS1DeltaE9 transgenic mouse. *Neurobiol. Dis.* 28 3–15. 10.1016/j.nbd.2007.06.015 17662610 PMC2245889

[B127] Pérez-Escuredo J. Van Hée V. F. Sboarina M. Falces J. Payen V. L. Pellerin L. (2016). Monocarboxylate transporters in the brain and in cancer. *Biochim. Biophys. Acta* 1863 2481–2497. 10.1016/j.bbamcr.2016.03.013 26993058 PMC4990061

[B128] Perry T. L. (1982). “Cerebral amino acid pools,” in *Handbook of Neurochemistry Chemical*, 2nd Edn, ed. Lajtha A. (New York, NY: Plenum Press), 151–180.

[B129] Pettegrew J. W. Klunk W. E. Panchalingam K. Kanfer J. N. McClure R. J. (1995). Clinical and neurochemical effects of acetyl-L-carnitine in Alzheimer’s disease. *Neurol. Aging* 16 1–4. 10.1016/0197-4580(95)80001-8 7723928

[B130] Pietrocola F. Galluzzi L. Bravo-San Pedro J. M. Madeo F. Kroemer G. (2015). Acetyl-CoA: central metabolite and second messenger. *Cell Metab.* 21 805–821. 10.1016/j.cmet.2015.05.014 26039447

[B131] Potter P. E. Rauschkolb P. K. Pandya Y. Sue L. I. Sabbagh M. N. Walker D. G. (2011). Pre- and post-synaptic cortical cholinergic deficits are proportional to amyloid plaque presence and density at preclinical stages of Alzheimer’s disease. *Acta Neuropathol.* 122 49–60. 10.1007/s00401-011-0831-1 21533854 PMC3362487

[B132] Prass R. L. Isohashi F. Utter M. F. (1980). Purification and characterization of an extramitochondrial acetyl coenzyme A hydrolase from rat liver. *J. Biol. Chem.* 255 5215–5223. 6102995

[B133] Rae C. Fekete A. D. Kashem M. A. Nasrallah F. A. Bröer S. (2012). Metabolism, compartmentation, transport and production of acetate in the cortical brain tissue slice. *Neurochem. Res.* 37 2541–2553. 10.1007/s11064-012-0847-5 22851350

[B134] Reijnierse G. L. Veldstra H. Van den Berg C. J. (1975). Short-chain fatty acid synthases in brain. Subcellular localization and changes during development. *Biochem. J.* 152 477–484. 10.1042/bj1520477 5995 PMC1172499

[B135] Rendina A. N. Cheng D. (2005). Characterization of the inactiviation of rat fatty acid synthase by C75: inhibition of partial reactions and protection by substrates. *Biochem. J.* 388 895–903. 10.1042/BJ20041963 15715522 PMC1183470

[B136] Rhein V. Song X. Wiesner A. Ittner L. M. Baysang G. Meier F. (2009). Amyloid-beta and tau synergistically impair the oxidative phosphorylation system in triple transgenic Alzheimer’s disease mice. *Proc. Natl. Acad. Sci. U.S.A.* 106 20057–20062. 10.1073/pnas.0905529106 19897719 PMC2774257

[B137] Rícný J. Tucek J. (1981). Acetyl coenzyme A and acetylcholine in slices of rat caudate nuclei incubated in the presence of metabolic inhibitors. *J. Biol. Chem.* 256 4919–4923. 7228860

[B138] Rícný J. Tucek S. (1982). Acetylcoenzyme A and acetylcholine in slices of rat caudate nuclei incubated with (-)hydroxycitrate, citrate and EGTA. *J. Neurochem.* 39 668–673. 10.1111/j.1471-4159.1982.tb07944.x 6808088

[B139] Rícný J. Tuèek S. (1983). Ca2 + ions and the output of acetylcoenzyme A from brain mitochondria. *Gen. Physiol. Biophys.* 2 27–37.

[B140] Rícný J. Tucek S. Nováková J. (1992). Acetylcarnitine, carnitine and glucose diminish the effect of muscarinic antagonist quinuclinyl benzilate on striatal acetylcholine content. *Brain Res.* 576 215–219. 10.1016/0006-8993(92)90683-Z 1515917

[B141] Ronowska A. Dyś A. Jankowska-Kulawy A. Klimaszewska-Łata J. Bielarczyk H. Romianowski P. (2010). Short-term effects of zinc on acetylcholine metabolism and viability of SN56 cholinergic neuroblastoma cells. *Neurochem. Int.* 56 143–151. 10.1016/j.neuint.2009.09.012 19781588

[B142] Ronowska A. Gul-Hinc S. Bielarczyk H. Pawełczyk T. Szutowicz A. (2007). Effects of zinc on SN56 cholinergic neuroblastoma cells. *J. Neurochem.* 103 972–983. 10.1111/j.1471-4159.2007.04786.x 17662047

[B143] Rossier J. Spantidakis Y. Benda P. (1977). The effect of Cl- on choline acetyltransferase kinetic parameters and a proposed role for Cl- in the regulation of acetylcholine synthesis. *J. Neurochem.* 29 1007–1012. 10.1111/j.1471-4159.1977.tb06504.x 599338

[B144] Rowlands B. D. Klugmann M. Rae C. D. (2017). Acetate metabolism does not reflect astrocytic activity, contributes directly to GABA synthesis, and is increased by silent information regulator 1 activation. *J. Neurochem.* 140 903–918. 10.1111/jnc.13916 27925207

[B145] Ryan R. McClure W. O. (1980). Physical and kinetic properties of choline acetyl transferase from rat and bovine brain. *J. Neurochem.* 34 395–403. 10.1111/j.1471-4159.1980.tb06609.x 7411145

[B146] Sawmiller D. R. Nguyen H. T. Markov O. Chen M. (2012). High-energy compounds promote physiological processing of Alzheimer’s amyloid-β precursor protein and boost cell survival in culture. *J. Neurochem.* 123 525–531. 10.1111/j.1471-4159.2012.07923.x 22906069

[B147] Schreiner S. J. Kirchner T. Narkhede A. Wyss M. Van Bergen J. M. G. Steininger S. C. (2018). Brain amyloid burden and cerebrovascular disease are synergistically associated with neurometabolism in cognitively unimpaired older adults. *Neurobiol. Aging* 63 152–161. 10.1016/j.neurobiolaging.2017.12.004 29310864

[B148] Schuberth J. Sollenberg J. Sundwall A. Sörbo B. (1966). Acetyl-CoA in brain. The effect of centrally active drugs, insulin coma and hypoxia. *J. Neurochem.* 13 819–822. 10.1111/j.1471-4159.1966.tb05877.x 5955048

[B149] Sensi S. L. Paoletti P. Bush A. I. Sekler I. (2009). Zinc in the physiology and pathology of the CNS. *Nat. Rev. Neurosci.* 10 780–791. 10.1038/nrn2734 19826435

[B150] Sharman E. H. Vaziri N. D. Ni Z. Sharman K. G. Bondy S. C. (2002). Reversal of biochemical and behavioral parameters of brain aging by melatonin and acetyl-L-carnitine. *Brain Res.* 957 223–230. 10.1016/S0006-8993(02)03551-5 12445964

[B151] Shea P. A. Aprison M. H. (1973). An enzymatic method for measuring picomole quantities of acetylcholine and choline in CNS tissue. *Anal. Biochem.* 56 165–177. 10.1016/0003-2697(73)90181-4 4358017

[B152] Shea P. A. Aprison M. H. (1977). The distribution of acetyl-CoA in specific areas of the CNS of the rat as measured by a modification of a radio-enzymatic assay for acetylcholine and choline. *J. Neurochem.* 28 51–58. 10.1111/j.1471-4159.1977.tb07707.x 833604

[B153] Shurubor Y. I. D’Aurelio M. Clark-Matott J. Isakova E. P. Deryabina Y. I. Beal M. F. (2017). Determination of coenzyme A and acetyl-coenzyme A in biological samples using HPLC with UV detection. *Molecules* 22:1388. 10.3390/molecules22091388 28832533 PMC6151540

[B154] Simpson I. A. Carruthers A. Vanucci S. (2007). Supply and demand in cerebral energy metabolism: the role of nutrient transporters. *J. Cereb. Blood Flow Metab.* 27 1766–1791. 10.1038/sj.jcbfm.9600521 17579656 PMC2094104

[B155] Sivanand S. Viney I. Wellen K. E. (2018). Spatiotemporal control of acetyl-CoA metabolism in chromatin regulation. *Trends Biochem. Sci.* 43 61–74. 10.1016/j.tibs.2017.11.004 29174173 PMC5741483

[B156] Srere P. A. (1965). The molecular physiology of citrate. *Nature* 205 766–770. 10.1038/205766a0

[B157] Sterling G. H. McCafferty M. R. O’Neil J. J. (1981). β-Hydroxybutyrate as precursor to acetyl moiety of acetycholine. *J. Neurochem.* 37 1250–1259. 10.1111/j.1471-4159.1981.tb04675.x 7028919

[B158] Suematsu N. Isohashi F. (2006). Molecular cloning and functional expression of human cytosolic acetyl-CoA hydrolase. *Acta Biochim. Polon.* 53 553–561. 16951743

[B159] Sun J. Pan C. Q. Chew T. W. Liang F. Burmeister M. Low B. C. (2015). BNIP-H recruits the cholinergic machinery to neurite terminals to promote acetylcholine signaling and neuritogenesis. *Dev. Cell.* 34 555–568. 10.1016/j.devcel.2015.08.006 26343454

[B160] Sun Y. Li T. Xie C. Zhang Y. Zhou K. Wang X. (2016). Dichloroacetate treatment improves mitochondrial metabolism and reduces brain injury in neonatal mice. *Oncotarget* 7 31708–31722. 10.18632/oncotarget.9150 27153546 PMC5077971

[B161] Szutowicz A. (1979). “Regional and developmental correlations between choline acetyl transferase and ATP-citrate lyase in rat brain,” in *Biological Aspects of Learning, Memory Formation and Ontogeny of the CNS*, eds Mathies H. Krug M. Popov N. (Berlin: Akademie Verlag), 489–499.

[B162] Szutowicz A. (2001). Aluminum, NO, and nerve growth factor neurotoxicity in cholinergic neurons. *J. Neurosci. Res.* 66 1009–1018. 10.1002/jnr.10040 11746431

[B163] Szutowicz A. Bielarczyk H. (1987). Elimination of CoASH interference from acetyl-CoA cycling assay by maleic anhydride. *Anal. Biochem.* 164 292–296. 10.1016/0003-2697(87)90495-7 3674377

[B164] Szutowicz A. Bielarczyk H. Gul S. Ronowska A. Pawełczyk T. Jankowska-Kulawy A. (2006). Phenotype-dependent susceptibility of cholinergic neuroblastoma cells to neurotoxic inputs. *Metab. Brain Dis.* 21 149–161. 10.1007/s11011-006-9007-4 16724269

[B165] Szutowicz A. Bielarczyk H. Gul S. Zieliński P. Pawełczyk T. Tomaszewicz M. (2005). Nerve growth factor and acetyl-L-carnitine evoked shifts in acetyl-CoA and cholinergic SN56 cell vulnerability to neurotoxic inputs. *J. Neurosci. Res.* 79 185–192. 10.1002/jnr.20276 15558747

[B166] Szutowicz A. Bielarczyk H. Jankowska-Kulawy A. Pawełczyk T. Ronowska A. (2013). Acetyl-CoA the key factor for survival or death of cholinergic neurons in course of neurodegenerative diseases. *Neurochem. Res.* 38 1523–1542. 10.1007/s11064-013-1060-x 23677775 PMC3691476

[B167] Szutowicz A. Bielarczyk H. Kisielevski Y. Jankowska A. Madziar B. Tomaszewicz M. (1998a). Effects of aluminium and calcium on acetyl-CoA metabolism in rat brain mitochondria. *J. Neurochem.* 71 2447–2453. 9832143 10.1046/j.1471-4159.1998.71062447.x

[B168] Szutowicz A. Bielarczyk H. Łysiak W. (1981). The role of citrate derived from glucose in the acetylcholine synthesis in rat brain synaptosomes. *Int. J. Biochem.* 13 887–892. 10.1016/0020-711X(81)90014-8 7274536

[B169] Szutowicz A. Bielarczyk H. Sosnowska D. Ciszek B. (1989). Regulation of citrate metabolism and acetycholine synthesis by Ca^2+^ in rat brain synaptosomes. *Neurochem. Int.* 15 403–409. 10.1016/0197-0186(89)90157-5 20504513

[B170] Szutowicz A. Bielarczyk H. Skulimowska H. (1994a). Effect of dichloroacetate on acetyl-CoA content and acetylcholine synthesis in rat brain synaptosomes. *Neurochem. Res.* 19 1107–1112. 10.1007/BF00965142 7824061

[B171] Szutowicz A. Bielarczyk H. Zyśk M. Dyś A. Ronowska A. Gul-Hinc S. (2017). Early and late pathomechanisms in Alzheimer’s disease. From zinc to amyloid-β neurotoxicity. *Neurochem. Res.* 42 891–904. 10.1007/s11064-016-2154-z 28039593 PMC5357490

[B172] Szutowicz A. Harris N. F. Srere P. A. Crawford I. L. (1983). ATP-citrate lyase and other enzymes of acetyl-CoA metabolism in fractions of small and large synaptosomes from rat brain hippocampus and cerebellum. *J. Neurochem.* 41 1502–1505. 10.1111/j.1471-4159.1983.tb00854.x 6137519

[B173] Szutowicz A. Kabata J. Łysiak W. (1980). ATP citrate lyase and other enzymes of acetyl-CoA metabolism in developing rat cerebrum and cerebellum. *Int. J. Biochem.* 11 545–549. 10.1016/0020-711X(80)90263-3 6103828

[B174] Szutowicz A. Łysiak W. (1980). Regional and subcellular distribution of ATP-citrate lyase and other enzymes of acetyl-CoA metabolism in rat brain. *J. Neurochem.* 35 775–785. 10.1111/j.1471-4159.1980.tb07073.x 6109001

[B175] Szutowicz A. Madziar B. Pawełczyk T. Tomaszewicz M. Bielarczyk H. (2004). Effects of NGF on acetylcholine, acetyl-CoA metabolism, and viability of differentiated and non-differentiated cholinergic neuroblastoma cells. *J. Neurochem.* 90 952–961. 10.1111/j.1471-4159.2004.02556.x 15287901

[B176] Szutowicz A. Stepień M. Bielarczyk H. Kabata J. Łysiak W. (1982). ATP citrate lyase in cholinergic nerve terminals. *Neurochem. Res.* 7 799–810. 10.1007/BF00965673 6126837

[B177] Szutowicz A. Tomaszewicz M. Bielarczyk H. Jankowska A. (1998b). Putative significance of shifts in acetyl-CoA compartmentalization in nerve terminals for disturbances of cholinergic transmission in brain. *Dev. Neurosci.* 20 485–492. 10.1159/000017347 9778588

[B178] Szutowicz A. Tomaszewicz M. Bielarczyk H. (1996). Disturbances of acetyl-CoA, energy and acetylcholine metabolism in some encephalopathies. *Acta Neurobiol. Exp.* 56 323–339. 10.55782/ane-1996-1137 8787193

[B179] Szutowicz A. Tomaszewicz M. Jankowska A. Kisielevski Y. (1994b). Acetylcholine synthesis in nerve terminals of diabetic rats. *Neuroreport* 5 2421–2424. 10.1097/00001756-199412000-00004 7696572

[B180] Szutowicz A. Stepień M. Łysiak W. Angielski S. (1976). Effect of (-)hydroxycitrate on the activities of ATP citrate lyase and enzymes of acetyl-CoA metabolism in rat brain. *Acta Biochim. Pol.* 23 227–234. 970036

[B181] Terwel D. Bothmer J. Wolf E. Meng F. Jolles J. (1998). Affected enzyme activities in Alzheimer’s disease are sensitive to antemortem hypoxia. *J. Neurol. Sci.* 161 47–56. 10.1016/S0022-510X(98)00240-8 9879681

[B182] Thevenet J. De Marchi U. Santo Domingo J. Christinat N. Bultot L. (2016). Medium-chain fatty acids inhibit mitochondrial metabolism in astrocytes promoting astrocyte-neuron lactate and ketone body shuttle systems. *FASEB J.* 30 1913–1926. 10.1096/fj.201500182 26839375

[B183] Tomaszewicz M. Bielarczyk H. Jankowska A. Szutowicz A. (1997). “Modification by nitric oxide of acetyl-CoA and acetylcholine metabolism in nerve terminals,” in *Neurochemistry Cellular, Molecular, and Clinical Aspects*, eds Teelken A. Korf J. (New York, NY: Plenum Press), 993–997.

[B184] Tomaszewicz M. Rossner S. Schliebs R. Ćwikowska J. Szutowicz A. (2003). Changes in cortical acetyl-CoA metabolism after selective basal forebrain cholinergic degeneration by 192IgG-saporin. *J. Neurochem.* 87 318–324. 10.1046/j.1471-4159.2003.01983.x 14511109

[B185] Triaca V. Calissano P. (2016). Impairment of the nerve growth factor pathway driving amyloid accumulation in cholinergic neurons: the incipit of the Alzheimer’s disease story? *Neural. Regen. Res.* 11 1553–1556. 10.4103/1673-5374.193224 27904476 PMC5116824

[B186] Trumble G. E. Smith M. A. Winder W. W. (1995). Purification and characterization of rat skeletal muscle acetyl-CoA carboxylase. *Eur. J. Biochem.* 231 192–198. 10.1111/j.1432-1033.1995.0192f.x 7628470

[B187] Trushina E. Nemutlu E. Zhang S. Christensen T. Camp J. Mesa J. (2012). Defects of mitochondrial dynamics and metabolomic signatures of evolving energetic stress in mouse models of familial Alzheimer’s disease. *PLoS One* 7:e32737. 10.1371/journal.pone.0032737 22393443 PMC3290628

[B188] Tuček S. (1983). “The synthesis of acetylcholine,” in *ito. Handbook if Neurochemistry* Vol. 4 ed. Lajtha A. (New York, NY: Plenum Press), 219–249.

[B189] Tuček S. (1985). Regulation of acetylcholine synthesis in the brain. *J. Neurochem.* 44 11–24. 10.1111/j.1471-4159.1985.tb07106.x 3880580

[B190] Tuček S. (1993). Short-term control of the synthesis of acetylcholine. *Prog. Biophys. Mol. Biol.* 60 59–69. 10.1016/0079-6107(93)90013-A 8480028

[B191] Valor L. M. Viosca J. Lopez-Atalaya J. P. Barco A. (2013). Lysine acetyltransferases CBP and p300 as therapeutic targets in cognitive and neurodegenerative disorders. *Curr. Pharm. Design* 19 5051–5064. 10.2174/13816128113199990382 23448461 PMC3722569

[B192] Veech R. L. Bradshaw P. C. Clarke K. Curtis W. Pawlosky R. King M. T. (2017). Ketone bodies mimic the life span extending properties of caloric restriction. *IUBMB Life* 69 305–314. 10.1002/iub.1627 28371201

[B193] Vossel K. A. Ranasinghe K. G. Beagle A. J. Mizurini D. Honma S. M. Dowling A. S. (2016). Incidence and impact of subclinical epileptiform activity in Alzheimer’s disease. *Ann. Neurol.* 80 858–870. 10.1002/ana.24794 27696483 PMC5177487

[B194] Waagepetersen H. S. Schousboe A. Sonnewald U. (2007). “Glutamine, glutamate, and GABA: metabolic aspects,” in *Handbook of Neurochemistry and Molecular Biology*, 3rd Edn, eds Oja S. S. Schousboe A. Saransaari P. (Berlin: Springer), 1–21.

[B195] Wang L. Guo L. Lu L. Sun H. Shao M. Beck S. J. (2016). Synaptosomal mitochondrial dysfunction in 5xFAD mouse model of Alzheimer’s disease. *PLoS One* 11:e0150441. 10.1371/journal.pone.0150441 26942905 PMC4778903

[B196] Wang P. Chen M. Yang Z. Yu T. Zhu J. Zhou L. (2017). Activation of pyruvate dehydrogenase activity by bidichloroacetate improves survival and neurologic outcomes after cardiac arrest in rats. *Shock* 49 704–711. 10.1097/SHK.0000000000000971 28846566

[B197] Wang Z. Leng Y. Wang J. Liao H. M. Bergman J. Leeds P. (2016). Tubastatin A, an HDAC6 inhibitor, alleviates stroke-induced brain infarction and functional deficits: potential roles of a α-tubulin acetylation and FGF-21 up-regulation. *Sci. Rep.* 6:19626. 10.1038/srep19626 26790818 PMC4726180

[B198] Wapenaar H. van der Wouden P. E. Groves M. R. Rotili D. Mai A. Dekker F. J. (2015). Enzyme kinetics and inhibition of histone acetyltransferase KAT8. *Eur. J. Med. Chem.* 105 289–296. 10.1016/j.ejmech.2015.10.016 26505788 PMC4871228

[B199] Webster S. J. Bachstetter A. D. Nelson P. T. Schmitt F. A. Van Eldik L. J. (2014). Using mice to model Alzheimer’s dementia: an overview of the clinical disease and the preclinical behavioral changes in 10 mouse models. *Front. Genet.* 5:88 10.3389/fgene.2014.00088 PMC4005958 24795750

[B200] Wellen K. E. Hatzivassiliou G. Sachdeva U. M. Bui T. V. Cross J. R. Thomson C. B. (2009). ATP-citrate lyase links cellular metabolism to histone acetylation. *Science* 324 1076–1080. 10.1126/science.1164097 19461003 PMC2746744

[B201] Williams L. R. Rylett R. J. (1990). Exogenous nerve growth factor increases the activity of high-affinity choline uptake and choline acetyltransferase in brain of Fisher 344 male rats. *J. Neurochem.* 55 1042–1049. 10.1111/j.1471-4159.1990.tb04594.x 2384747

[B202] Wlassics I. D. Stille C. Anderson V. E. (1988). Coenzyme A dithioesters: synthesis, characterization and reaction with citrate synthase and acetyl-CoA:choline *O*-acetyltransferase. *Biochim. Biophys. Acta* 952 269–276. 10.1016/0167-4838(88)90126-4 3337828

[B203] Wohnsland S. Burgers H. F. Kuschinsky W. Maurer M. H. (2010). Neurons and neuronal stem cells survive in glucose-free lactate and in high glucose cell culture medium during normoxia and anoxia. *Neurochem. Res.* 35 1635–1642. 10.1007/s11064-010-0224-1 20602256

[B204] Wong V. S. C. Picci C. Swift M. Levinson M. Willis D. Langley B. (2018). α-Tubulin acetyltransferase is a novel target mediating neurite growth inhibitory effects of chondroitin sulfate proteoglycans and myelin-associated glycoprotein. *eNeuro* 5:ENEURO.0240-17.2018. 10.1523/ENEURO.0240-17.2018 29497702 PMC5830348

[B205] Yao J. Irwin R. W. Zhao L. Nilsen J. Hamilton R. T. Brinton R. D. (2009). Mitochondrial bioenergetics deficit precedes Alzheimer’s pathology in female mouse model of Alzheimer’s disease. *Proc. Natl. Acad. Sci. U.S.A.* 106 14670–14675. 10.1073/pnas.0903563106 19667196 PMC2732886

[B206] Yin J. X. Maalouf M. Han P. Zhao M. Gao M. Dharshaun T. (2016). Ketones block amyloid entry and improve cognition in an Alzheimer’s model. *Neurobiol. Aging* 39 25–37. 10.1016/j.neurobiolaging.2015.11.018 26923399

[B207] Zhang Y. Hong Y. Bounhar Y. Blacker M. Roucou X. Tounekti O. (2003). p75 neurotrophin receptor protects primary cultures of human neurons against extracellular amyloid beta peptide cytotoxicity. *J. Neurosci.* 23 7385–7394. 10.1523/JNEUROSCI.23-19-07385.2003 12917374 PMC6740455

[B208] Zhang Z. G. Wang X. Zai J. H. Sun C. H. Yan B. C. (2018). Icariin improves cognitive impairment after traumatic brain injury by enhancing hippocampal acetylation. *Chin. J. Integr. Med.* 2018 1–6. 10.1007/s11655-018-2823-z 29327125

[B209] Zhao S. Torres A. Henry R. A. Trefely S. Wallace M. Lee J. V. (2016). ATP-citrate lyase controls a glucose-to-acetate metabolic switch. *Cell Rep.* 17 1037–1052. 10.1016/j.celrep.2016.09.069 27760311 PMC5175409

[B210] Zhong X. Shi H. Shen Z. Hou L. Luo X. Chen X. (2014). 1H-proton magnetic resonance spectroscopy differentiates dementia with Lewy bodies from Alzheimer’s disease. *J. Alzheimer’s Dis.* 40 953–966. 10.3233/JAD-131517 24531156

[B211] Zhou Q. Lam P. Y. Han D. Cadenas E. (2009). Activation of c-Jun-N-terminal kinase and decline of mitochondrial pyruvate dehydrogenase activity during brain aging. *FEBS Lett.* 583 1132–1140. 10.1016/j.febslet.2009.02.043 19272379 PMC2672565

[B212] Zimatkin S. M. Oganesian N. A. Kiselevski Y. V. Deitrich R. A. (2011). Acetate-dependent mechanisms of inborn tolerance to ethanol. *Alcohol Alcohol.* 46 233–238. 10.1093/alcalc/agr014 21349883 PMC3114548

[B213] Zyśk M. Bielarczyk H. Gul-Hinc S. Dyś A. Gapys B. Ronowska A. (2017). Phenotype-dependent interactions between N-acetyl-L-aspartate and acetyl-CoA in septal SN56 cholinergic cells exposed to excess of zinc. *J. Alzheimer’s Dis.* 56 1145–1158. 10.3233/JAD-160693 28106547

